# Supplementary figures and images for: Scientific modelling can be accessible, interoperable and user friendly: A case study for pasture and livestock modelling in Spain
Source: PLoS One. 2023 Feb 24;18(2):e0281348. doi: 10.1371/journal.pone.0281348 (PMC9957615; doi:10.1371/journal.pone.0281348)

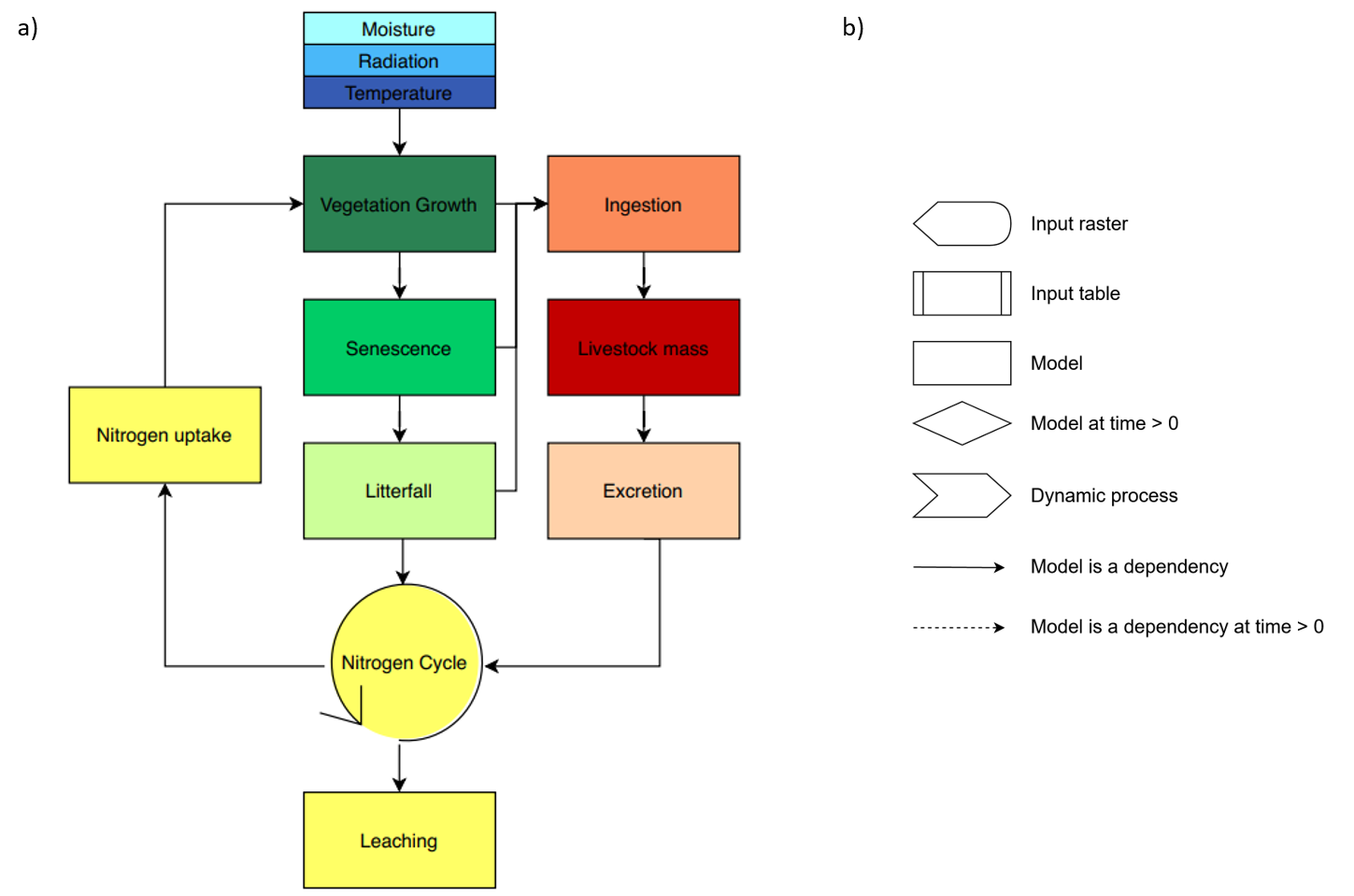

Supplement: S1 Fig — A) Namespace dataflow and B) Model’s dataflow legend. (TIF) [file pone.0281348.s001.tif]

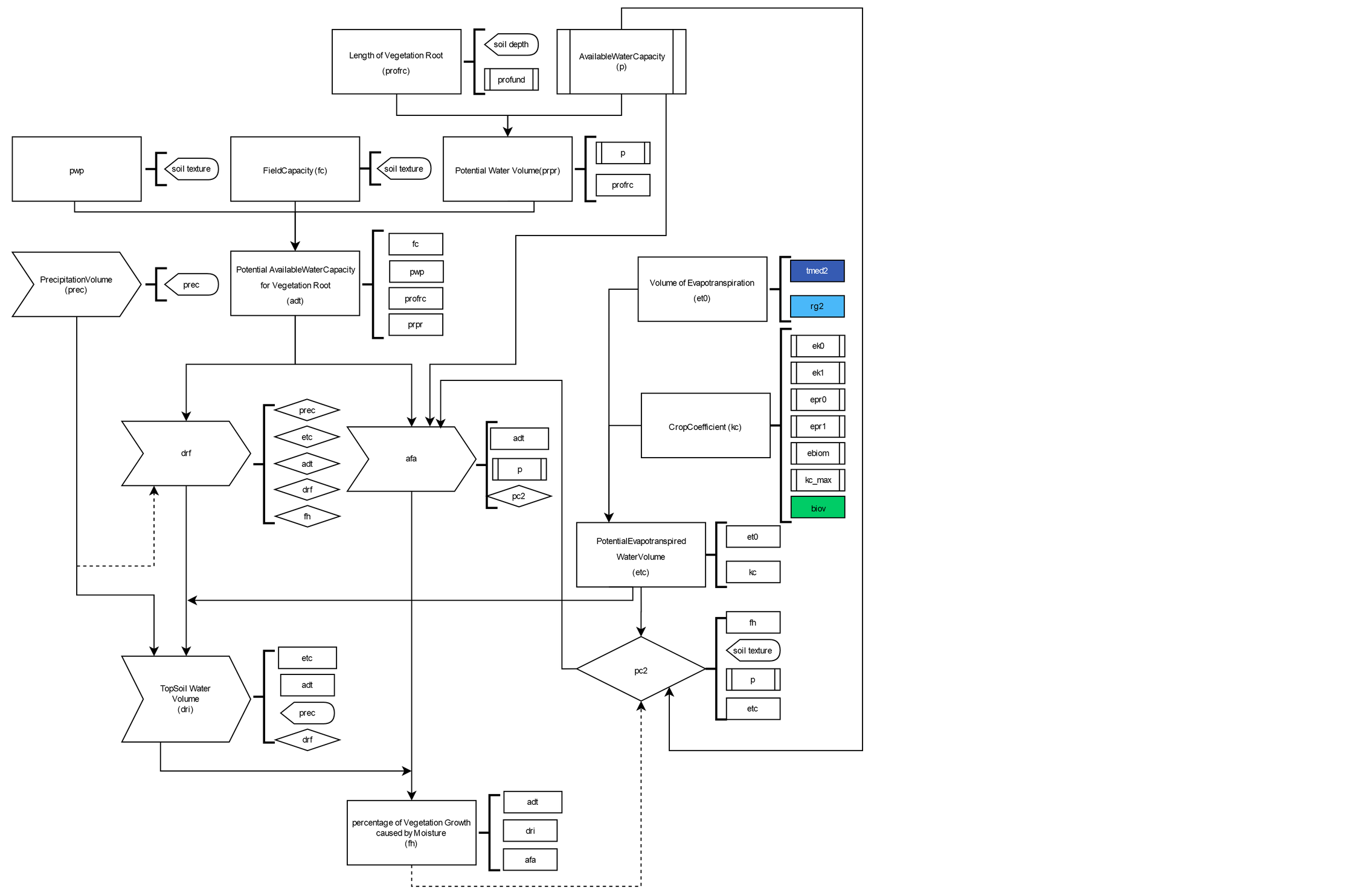

Supplement: S2 Fig — (TIF) [file pone.0281348.s002.tif]

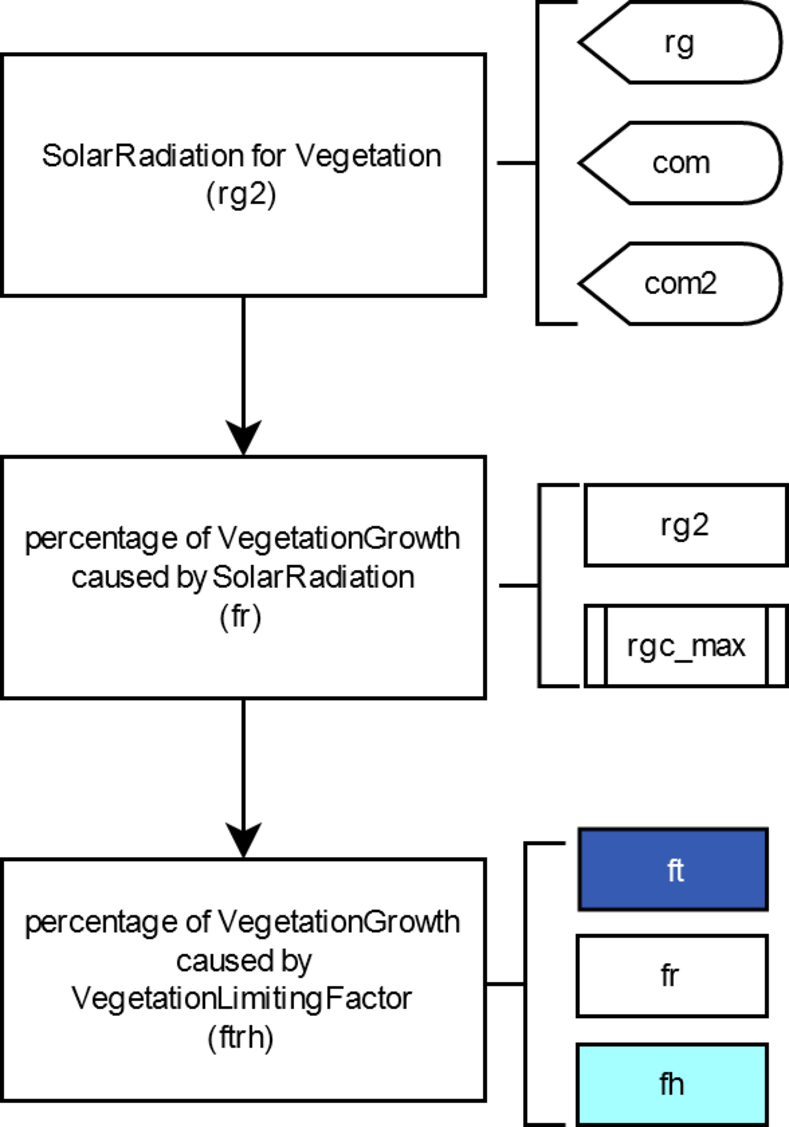

Supplement: S3 Fig — (TIF) [file pone.0281348.s003.tif]

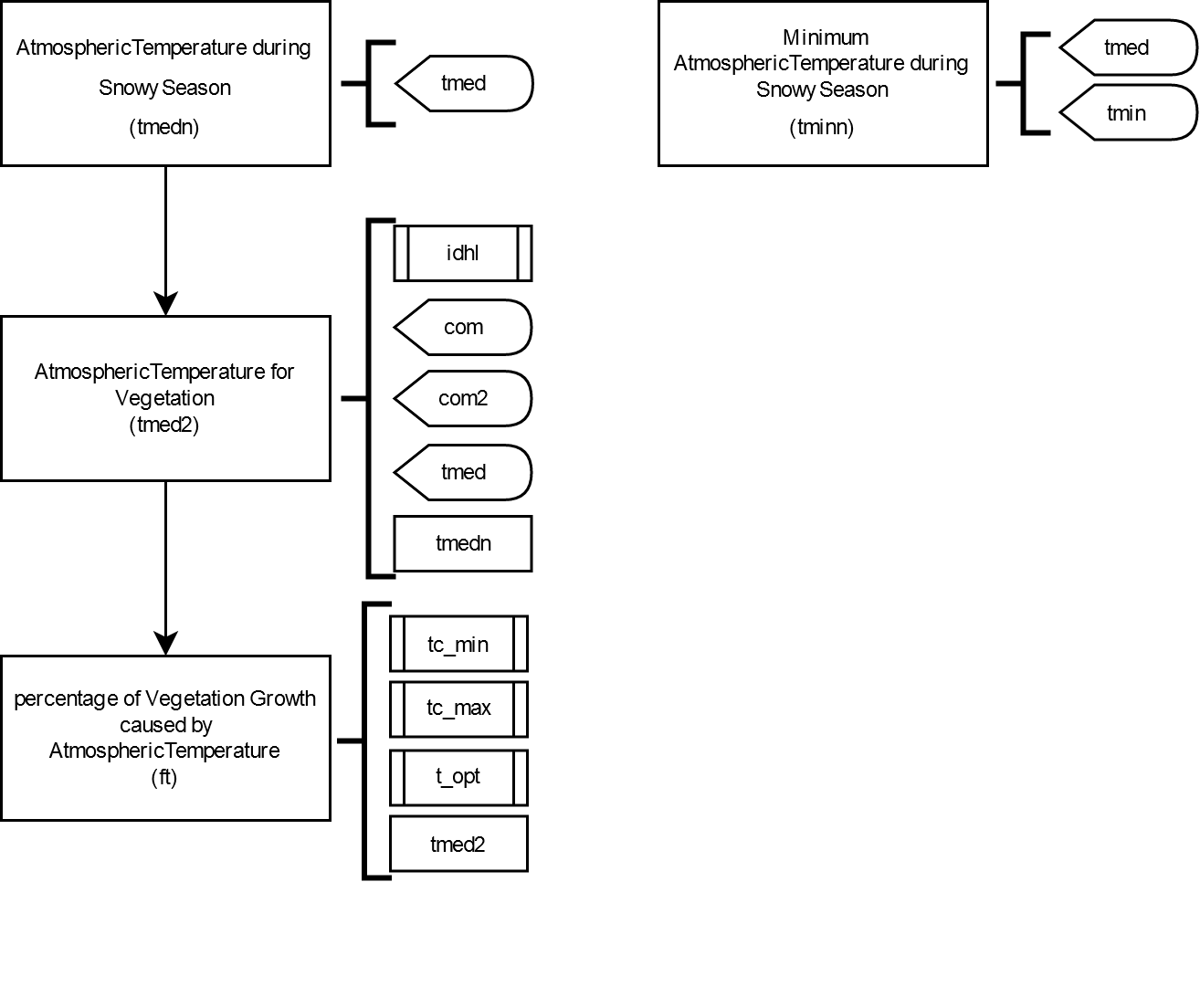

Supplement: S4 Fig — (TIF) [file pone.0281348.s004.tif]

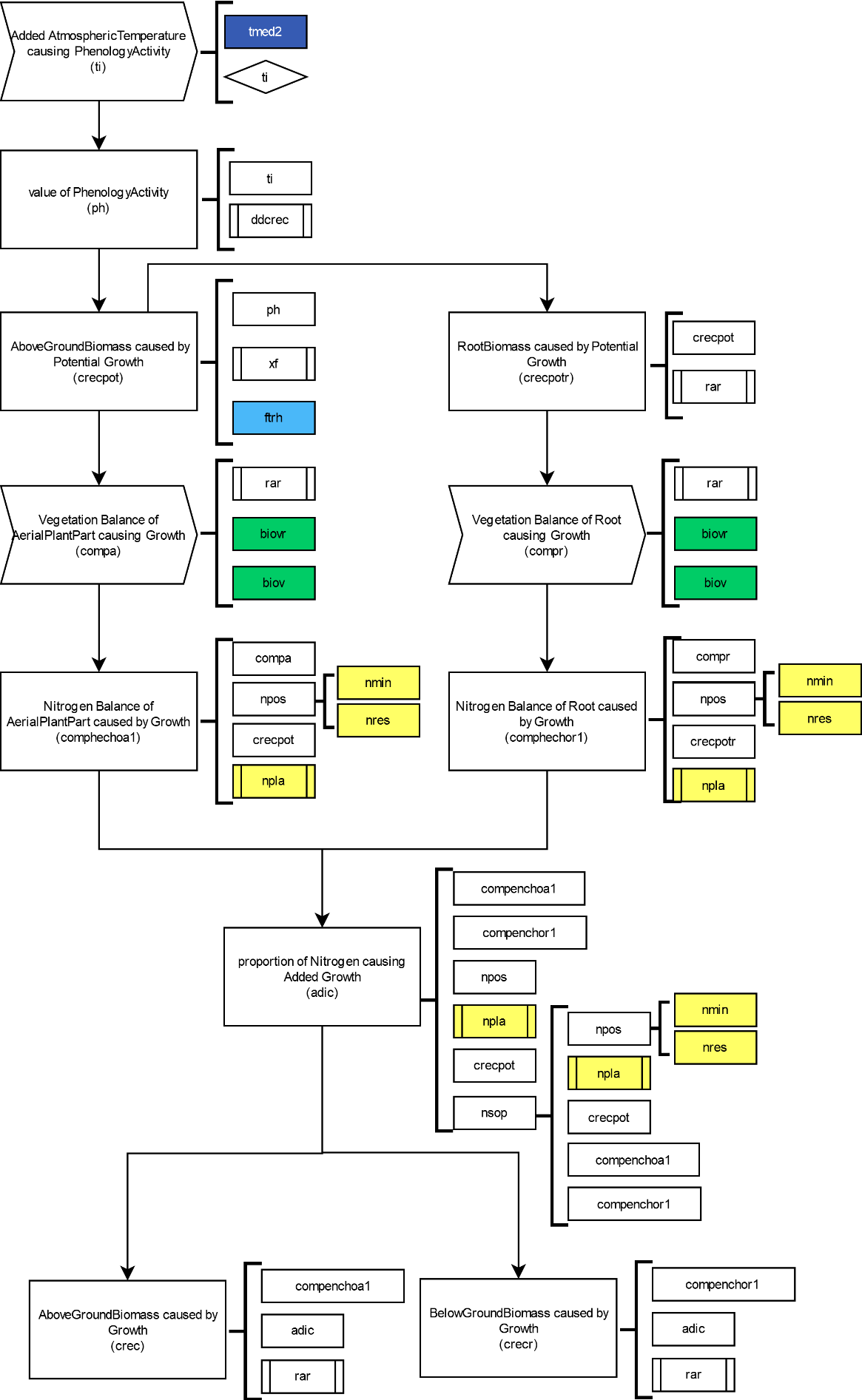

Supplement: S5 Fig — (TIF) [file pone.0281348.s005.tif]

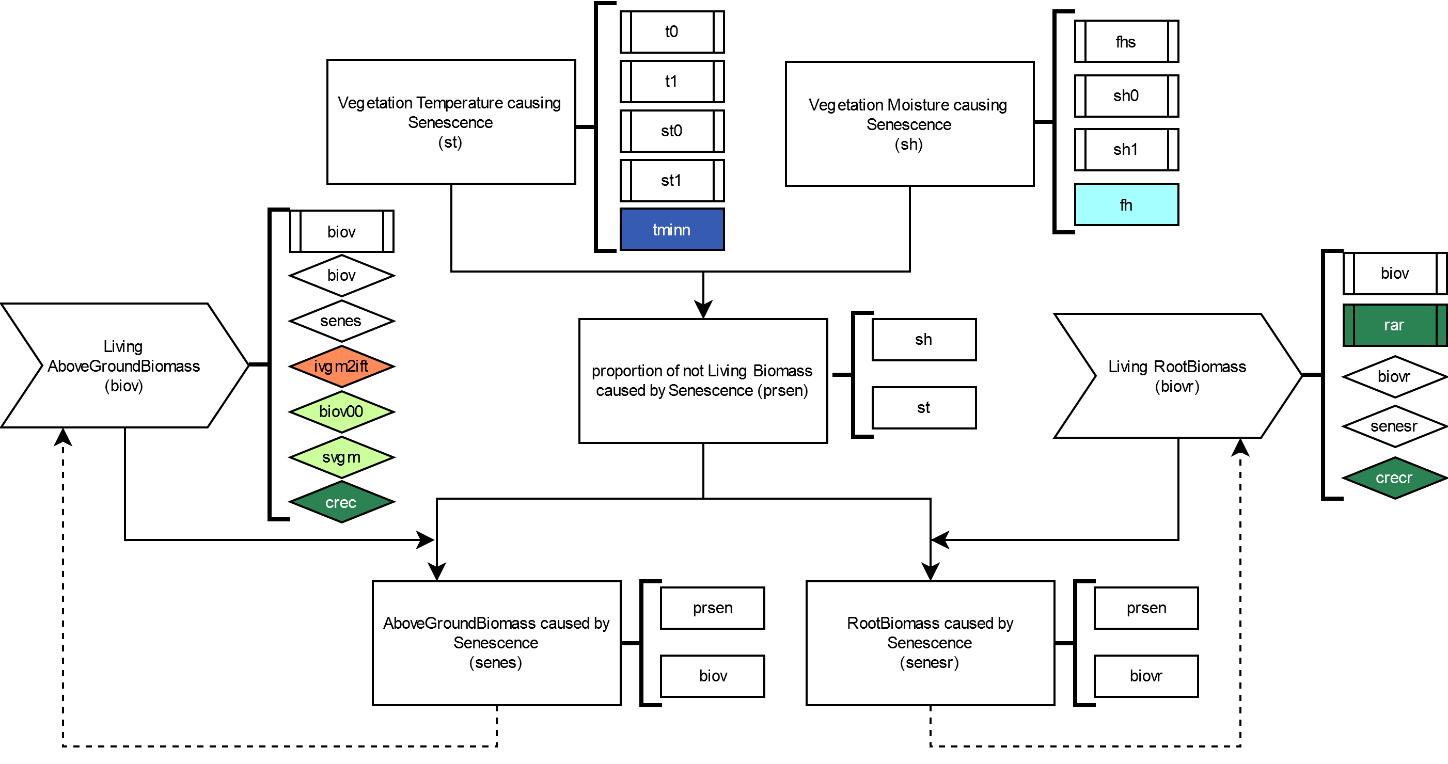

Supplement: S6 Fig — (TIF) [file pone.0281348.s006.tif]

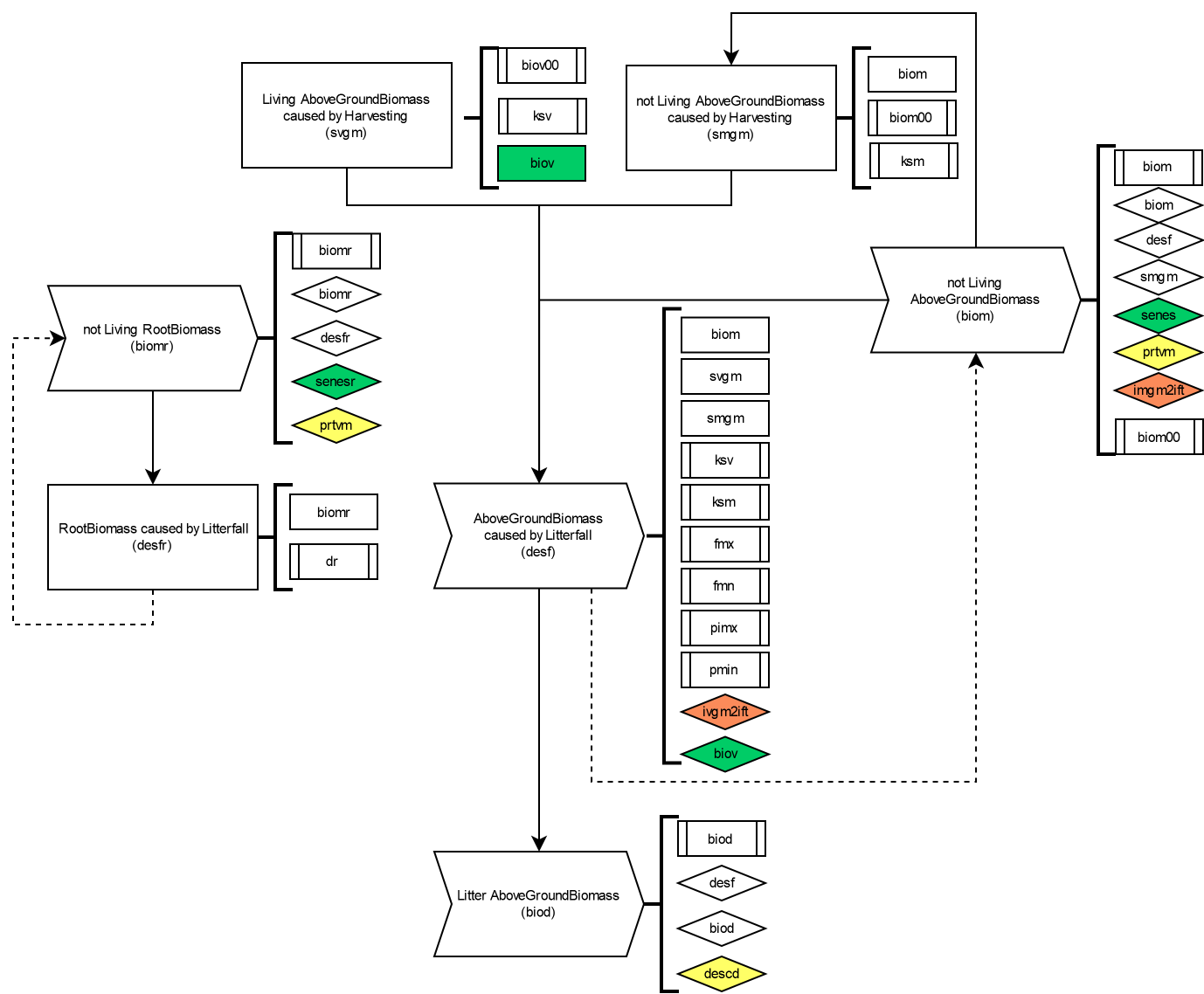

Supplement: S7 Fig — (TIF) [file pone.0281348.s007.tif]

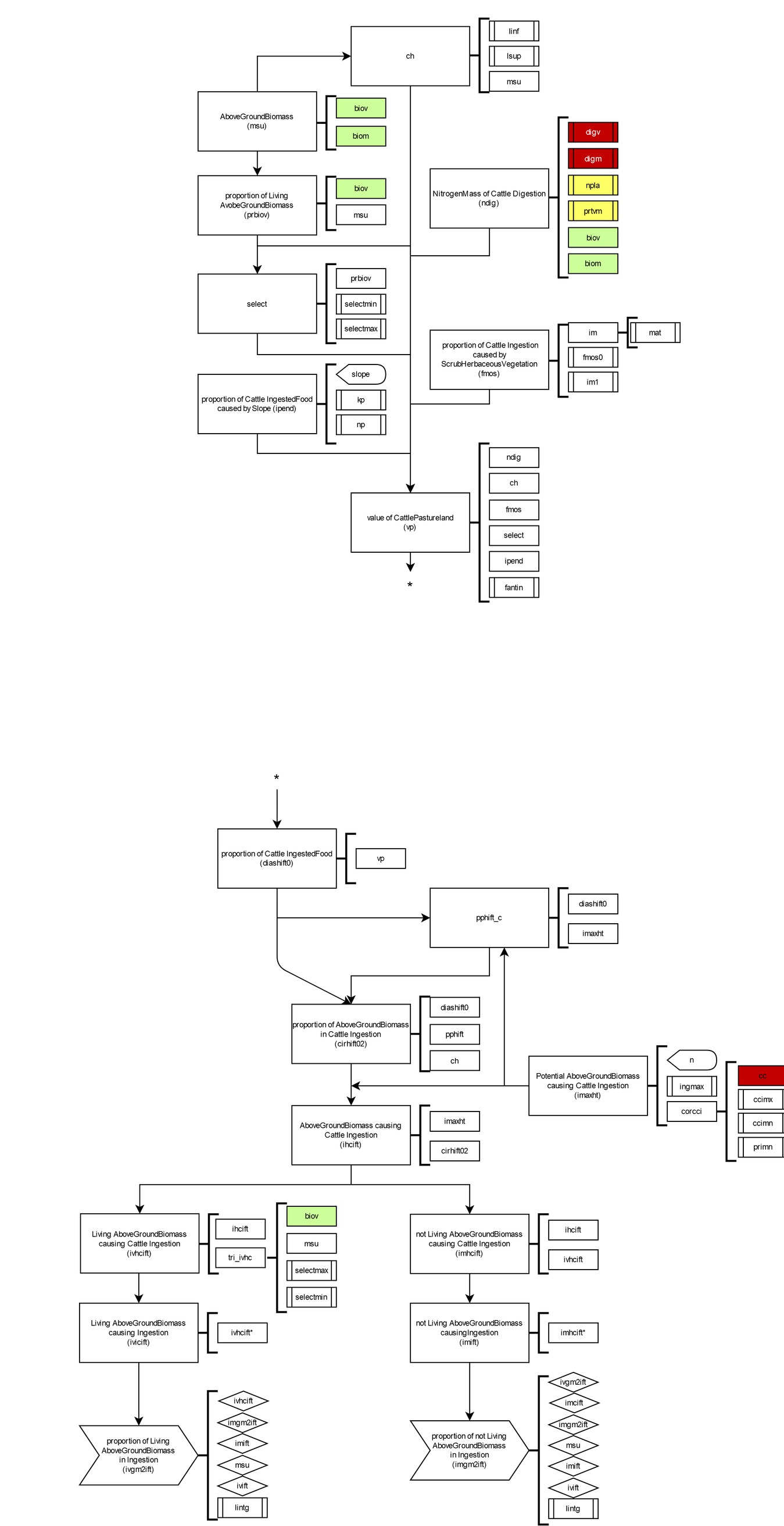

Supplement: S8 Fig — (TIF) [file pone.0281348.s008.tif]

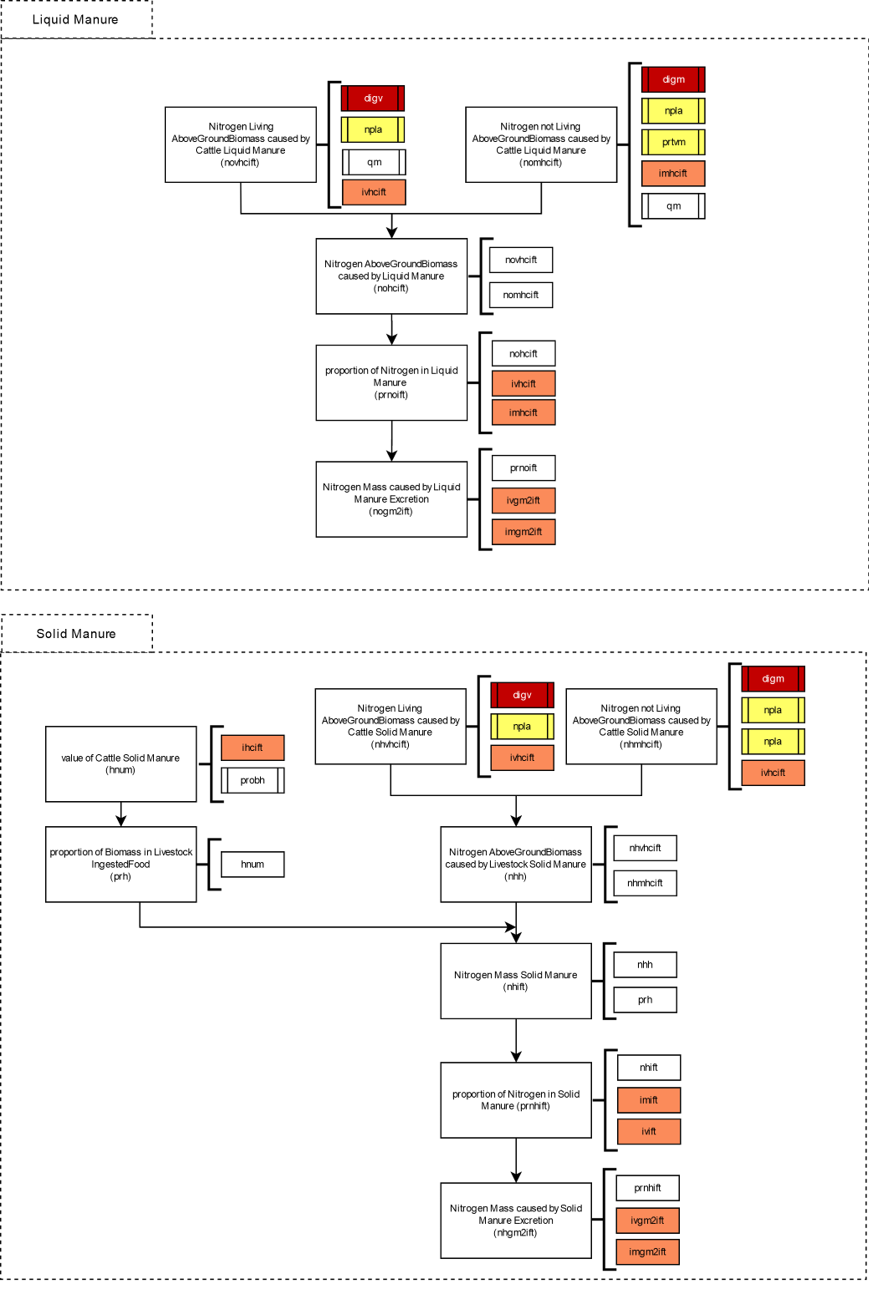

Supplement: S9 Fig — (TIF) [file pone.0281348.s009.tif]

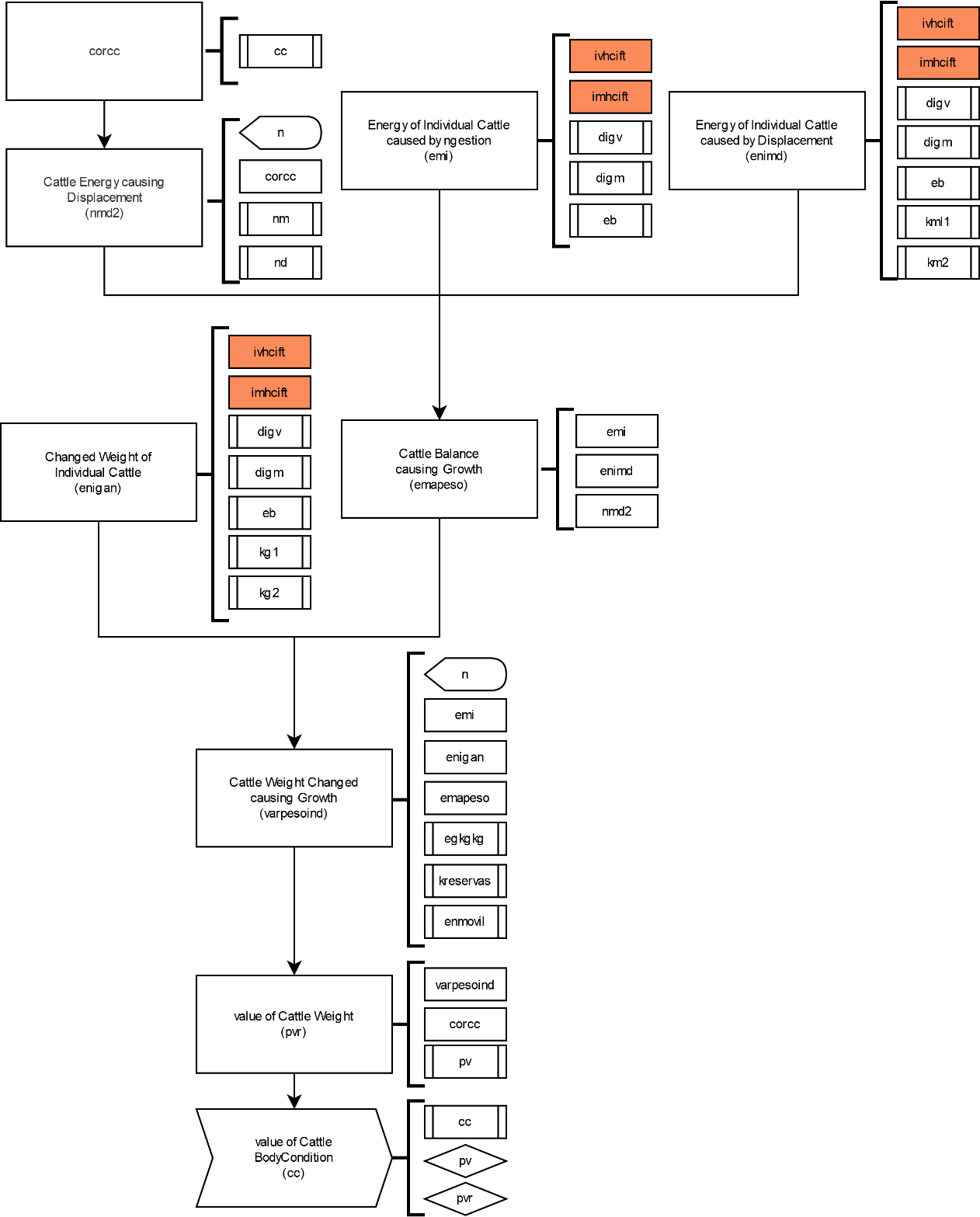

Supplement: S10 Fig — (TIF) [file pone.0281348.s010.tif]

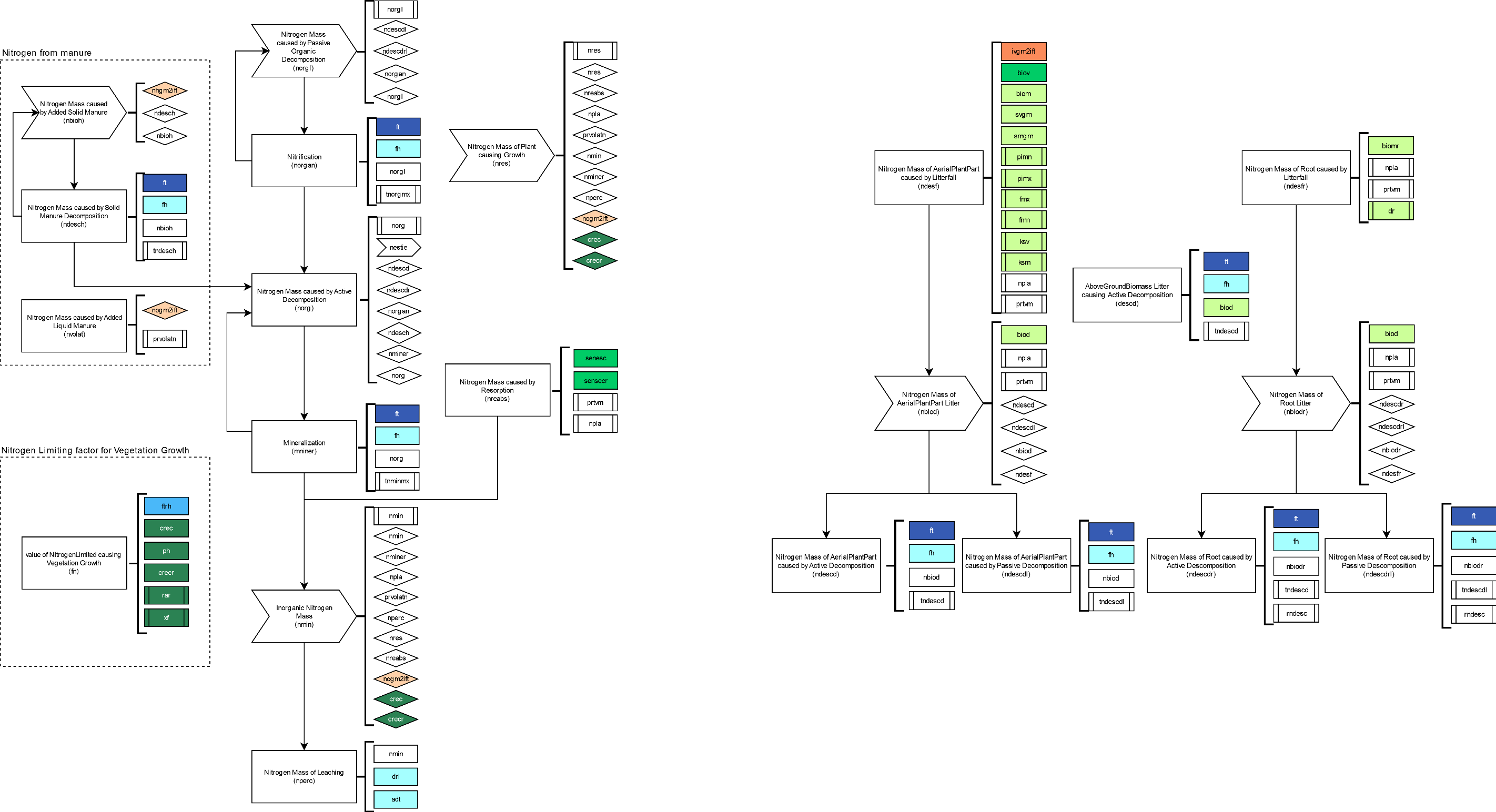

Supplement: S11 Fig — A. Dataflow of nitrogen cycle namespace. B. Dataflow of nitrogen cycle namespace. (TIF) [file pone.0281348.s011.tif]

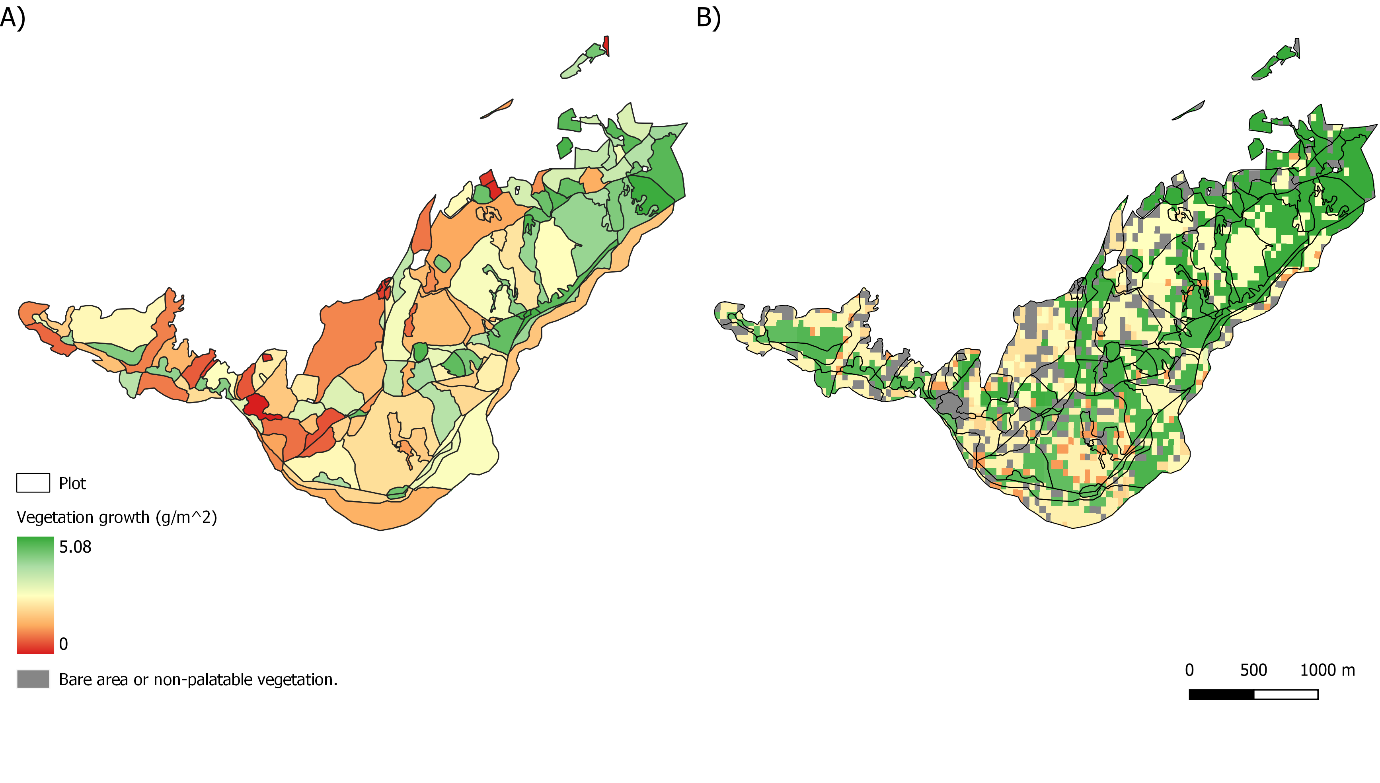

Supplement: S12 Fig — A) Puerto image and B) PaL map. (TIF) [file pone.0281348.s012.tif]

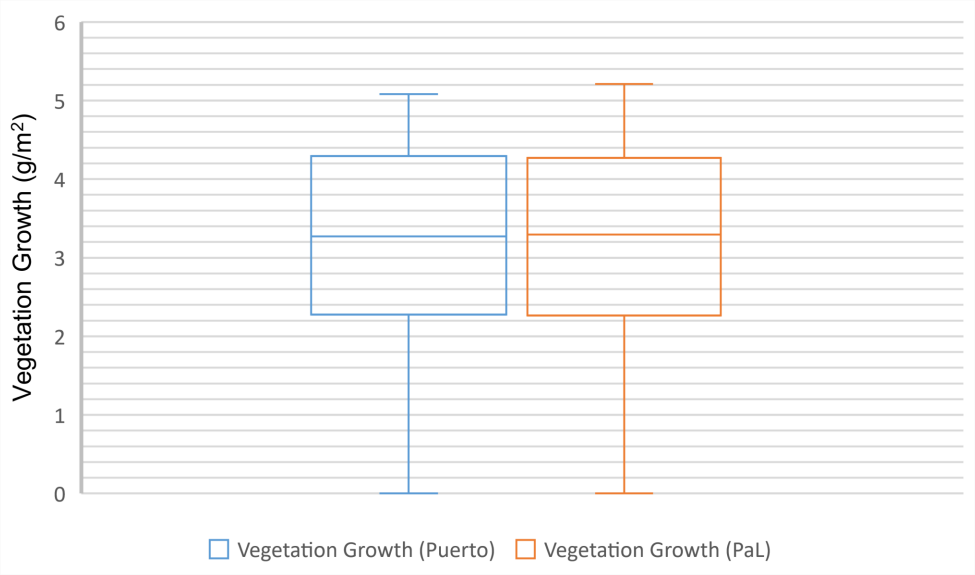

Supplement: S13 Fig — (TIF) [file pone.0281348.s013.tif]

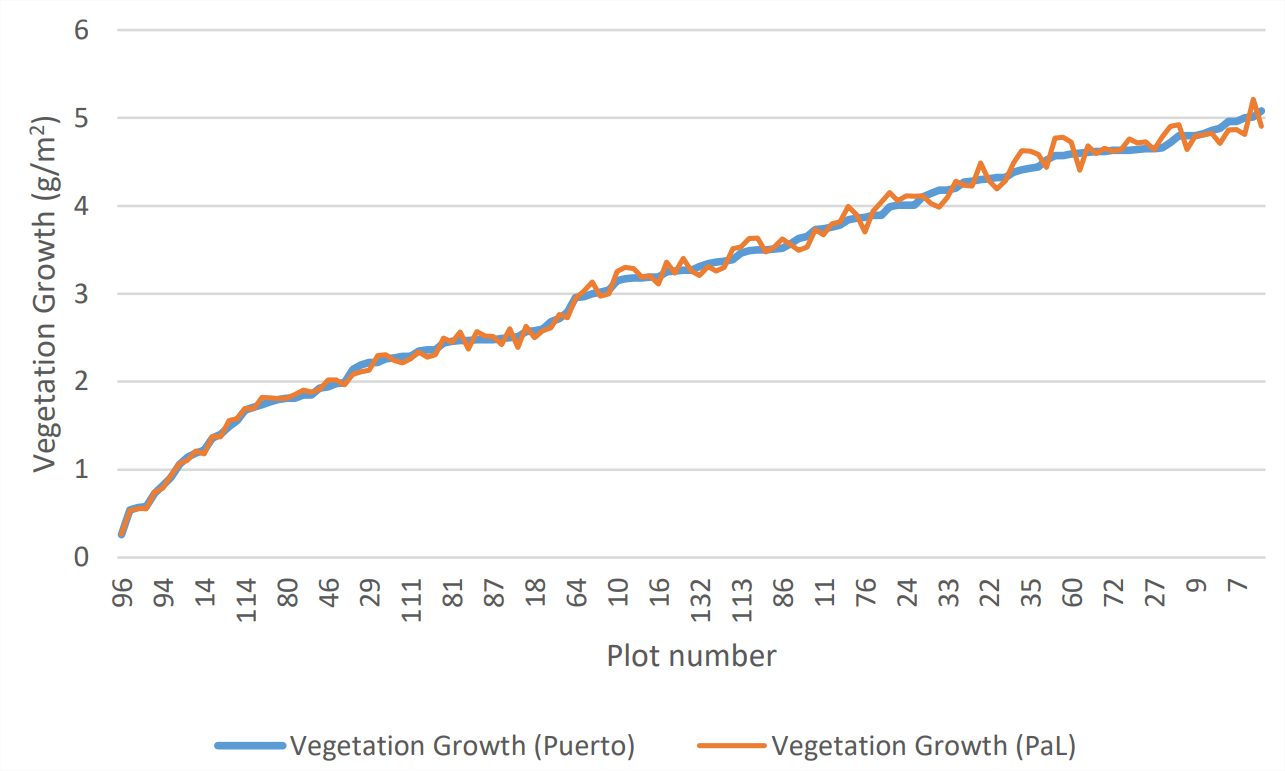

Supplement: S14 Fig — (TIF) [file pone.0281348.s014.tif]
