# Supplementary material for: Scientific modelling can be accessible, interoperable and user friendly: A case study for pasture and livestock modelling in Spain
Source: PLoS One. 2023 Feb 24;18(2):e0281348. doi: 10.1371/journal.pone.0281348 (PMC9957615; doi:10.1371/journal.pone.0281348)
Supplement: S1 File — (DOCX) [file pone.0281348.s015.docx]

**Supporting Information**

S1 Appendix

The Supporting Information S1 Appendix details dataflow, parameters and equations of the PaL model which have been adapted from Puerto model. Although all models are interoperable with them, they have been structured in namespaces. The general dataflow (S1 Fig.A) differentiates each of these namespaces by color. The color palette is applied to the dataflow of each namespace when a model runs in a namespace other than its own. The elements composing the dataflow follow the legend explained in S1 Fig.B.

General Model


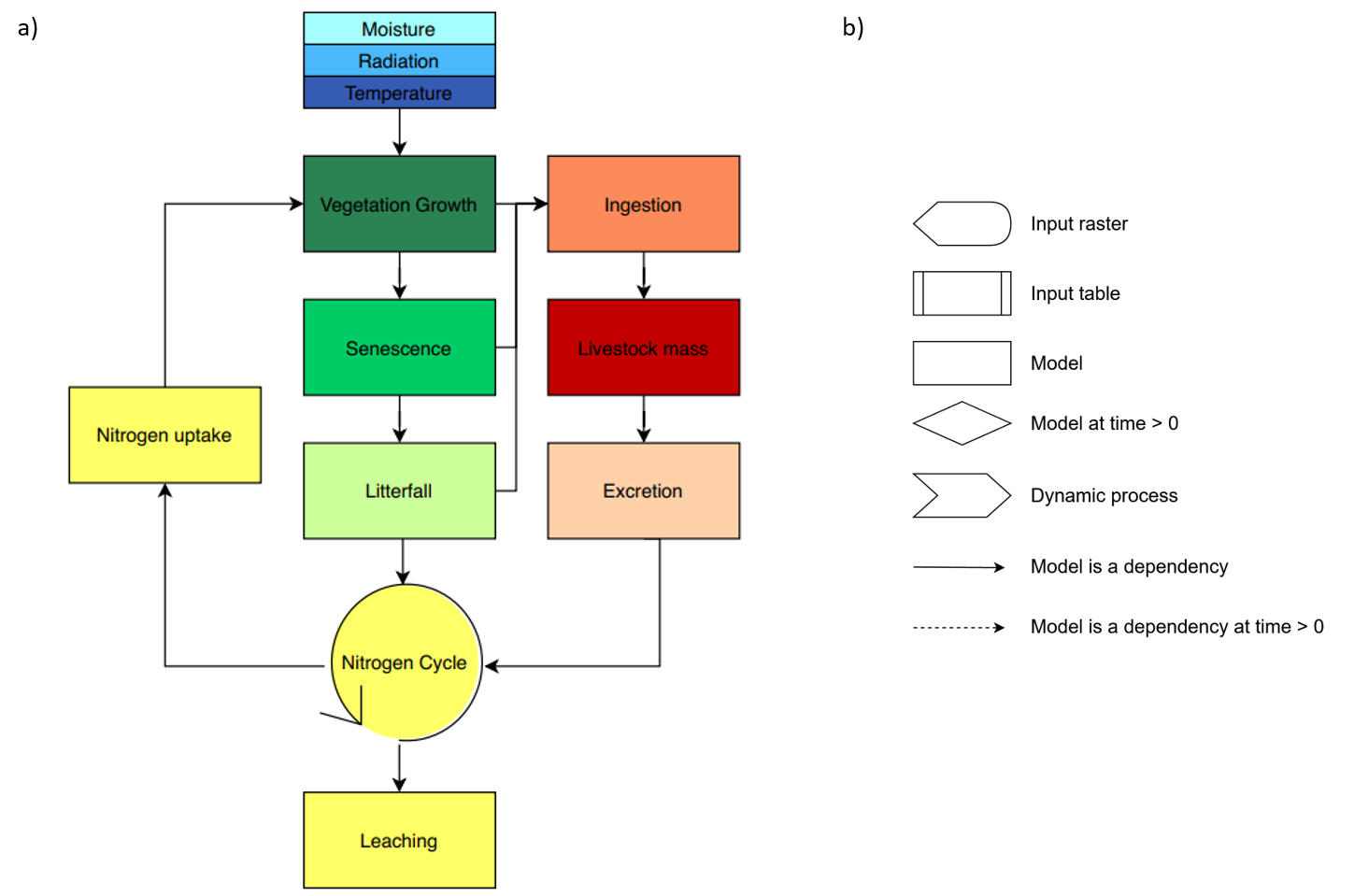


S1 Fig. General Information. A) Namespace dataflow and B) Model’s dataflow legend.

S1 Table Description of general models.

| **Puerto id** | **Semantic Model (k.IM language)** | **Description** | **Units** |
| --- | --- | --- | --- |
| com | Type of Relevant Gramineae Plant | Primary plant species |  |
| com2 | Type of Overstory Relevant Gramineae Plant | Secondary plant species |  |
| n_c | Count of Cattle Individual | Number of animals per hectare | individuals/ha |
| n_m | Count of Mares Individual | Number of animals per hectare | individuals/ha |

Moisture


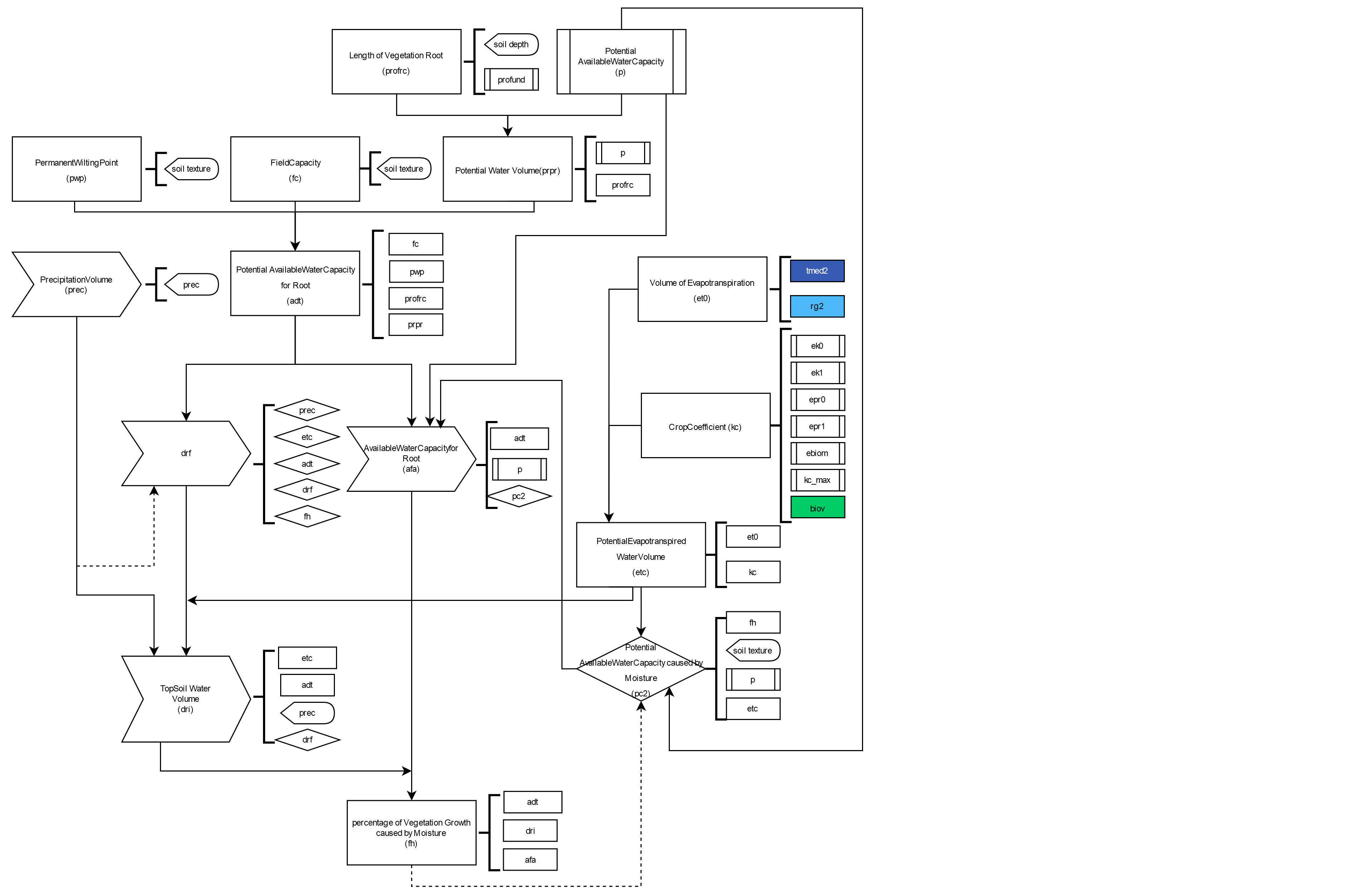


S2 Fig. Dataflow of moisture namespace.

S2A Table Description of moisture namespace models.

| **Puerto id** | **Semantic Model (k.IM language)** | **Description** | **Units** |
| --- | --- | --- | --- |
| adt | Potential AvailableWaterCapacity for Root | Potential available soil water in the root zone | mm |
| afa | AvailableWaterCapacity for Root | The readily available soil water in the root zone | mm |
| drf |  | Initial depletion |  |
| dri | TopSoil Water Volume | Volume of water in the root zone caused by the limits on root zone depletion by evapotranspiration | mm |
| ebiom |  | Biomass when Kc (crop coefficient) is 1 | g/m^2^ |
| ek0 |  | Minimum and constant Crop Coefficient |  |
| ek1 |  | Definition of the function slope between Kc (Crop coefficient) and biov (living biomass) | [0-1] |
| epr0 |  | Proportion of the biov (living biomass) reference below which Kc (Crop Coefficient) is minimum and constant (ek0) | [0-1] |
| epr1 |  | Proportion of the biov (living biomass) reference at which kc (Crop Coefficient) = kcmax (Maximum Crop coefficient) | [0-1] |
| et0 | Volume of Evapotranspiration | Reference evapotranspiration | mm |
| etc | PotentialEvapotranspiredWaterVolume | Potential Evapotranspiration | mm |
| fc | FieldCapacity | Field Capacity | m^3^/ m^3^ |
| prec | PrecipitationVolume | Volume of precipitation | mm |
| fh | Percentage of VegetationGrowth caused by Moisture | Coefficient between 0 and 1 that calculates vegetation water stress through soil moisture and vegetation characteristics | [0-1] |
| kc | CropCoefficient | Crop coefficient |  |
| kcmax |  | Maximum value of crop coefficient following rain or irrigation |  |
| p | Potential AvailableWaterCapacity | Average fraction of total available soil water that can be depleted from the root zone before moisture stress (reduction in ET) occurs | [0-1] |
| pc2 | Potential AvailableWaterCapacity caused by Moisture | Adjustment of p (AvailableWaterCapacity) for different etc (PotentialEvapotranspiredWaterVolume) | [0-1] |
| soil_depth | SoilDepth | Soil depth | mm |
| prec | PrecipitationVolume | Precipitation | mm |
| profr | Maximum Length of Root | Root depth when there are no bedrock limitations | mm |
| profrc | Length of Vegetation Root | Root depth considering limitations | mm |
| prpr | Potential Water Volume | Proportion of soil water content available to roots | [0-1] |
| pwp | PermanentWiltingPoint | Permanent wilting point, defined as the minimal amount of water in the soil that the plant requires to avoid wilting | m^3^/ m^3^ |
| soil_texture | Type of SoilTexture | Soil texture (sand, loam or clay) |  |

S2B Table Parameters of moisture namespace.

| com | ebiom | profr | kc_max | epr_0 | epr_1 | ek_0 | ek_1 | p |
| --- | --- | --- | --- | --- | --- | --- | --- | --- |
| Calluna (Calluna vulgaris) | 300 | 600 | 1.3 | 0.2 | 0.5 | 0.4 | 0.5 | 0.5 |
| FestucaRubra (Agrostis curtisii) | 250 | 300 | 1.3 | 0.2 | 0.5 | 0.4 | 0.5 | 0.5 |
| Helictotrichon (Helictotrichon cantabricum) | 250 | 500 | 1.3 | 0.2 | 0.5 | 0.4 | 0.5 | 0.5 |
| Phragmites | 250 | 300 | 1.3 | 0.2 | 0.5 | 0.4 | 0.5 | 0.5 |
| Polypodiopsida | 300 | 500 | 1.3 | 0.2 | 0.5 | 0.4 | 0.5 | 0.5 |
| Senecio (sp) | 250 | 300 | 1.3 | 0.2 | 0.5 | 0.4 | 0.5 | 0.5 |
| UlexGallii | 250 | 600 | 1.3 | 0.2 | 0.5 | 0.4 | 0.5 | 0.5 |
| Carex | 250 | 400 | 1.3 | 0.2 | 0.5 | 0.4 | 0.5 | 0.5 |
| Gramineae | 250 | 300 | 1.3 | 0.2 | 0.5 | 0.4 | 0.5 | 0.5 |
| UlexEuropaeus | 250 | 1000 | 1.3 | 0.2 | 0.5 | 0.4 | 0.5 | 0.5 |

S2C Table Equations of moisture namespace.

| id | time (t=>0) | |
| --- | --- | --- |
|  | **t=0** | **t>0** |
| kc | *kca=*  -1*ek1*epr0+ek0*epr1epr1-epr0  *kcb=*  ek1-ek0epr1-epr0*ebiom  if biov < epr0*ebiom {  ek0  } else if (kca+kcb* biov)> kc_max {  kc_max  } else {  kca+ kcb*biov } | |
| et0 | 0.0135 * tmed2 + 17.8* rg22.45 | |
| profrc | if soil_depth< profr{  soil_depth  }else{  profr | |
| prpr | profrc * p | |
| fc | if (soil_texture== Sand)  {  0.24  } else if (soil_texture== Loam) {  0.35  } else {  0.44 } | |
| pwp | if soil_texture== Sand {  0.09  } else if (soil_texture== Loam) {  0.16  } else {  0.26 } | |
| adt | (fc-pwp)*profrc*prpr | |
| etc | et0*kc | |
| afa | adt*p | adt*pc2 |
| pc2 | *a =*  p + 0.04 * (5-(fh*etc))  if soil_texture== Loam {  a  } else if soil_texture== Sand {  pc - 0.075 * pc  } else {  pc + 0.075 * pc } | |
| drf | 0 | *a =*  fh * etc - prec + drf  if a<0 {  0  } else if (a> adt) {  adt  } else {  a } |
| dri | *a =*  etc - prec  if a<0 {  0  } else if (a> adt) {  adt  } else {  a } | fh*etc-prec+drf |
| fh | if adt-driadt-afa>1 {  1  } else {  adt-driadt-afa | |

Radiation


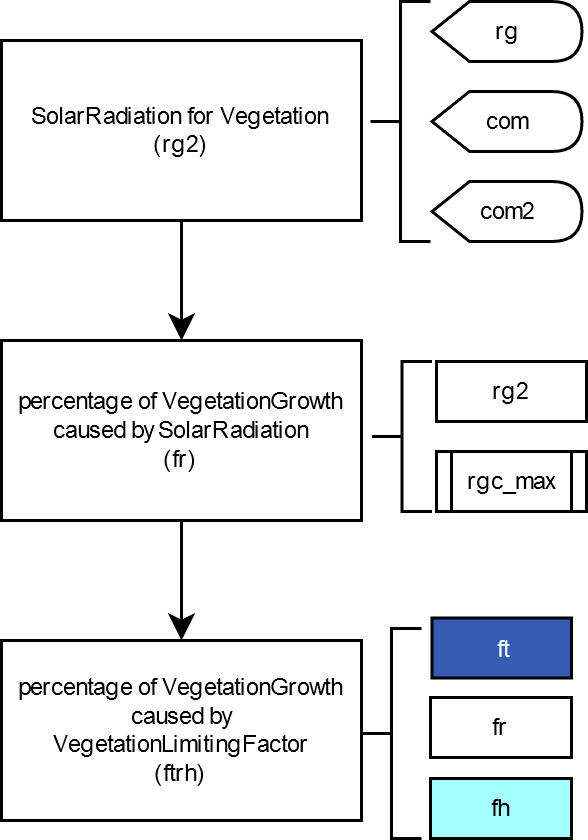


S3 Fig. Dataflow of radiation namespace.

S3A Table Description of radiation namespace models.

| **Puerto id** | **Semantic Model (k.IM language)** | **Description** | **Units** |
| --- | --- | --- | --- |
| **fr** | Percentage of Vegetation Growth caused by SolarRadiation | Percentage of radiation that limits vegetation growth. 0: No growth (maximum limitation), 1: Maximum growth (no limitation) | [0-1] |
| **ftrh** | Percentage of Vegetation Growth caused by VegetationLimitingFactor | Set of climatic factors (temperature, radiation and soil moisture) limiting vegetation growth | [0-1] |
| **rg** | SolarRadiation | Incidence of solar radiation | MJ/m^2^ |
| **rg2** | SolarRadiation for Vegetation | Incidence of solar radiation over vegetation | MJ/m^2^ |
| **rgc_max** | Maximum PhotosyntheticallyActiveRadiation | Incidence of solar radiation for maximum photosynthetic capacity | MJ/m^2^ |

S3B Table Parameters of radiation namespace.

| **com** | **rgc_max (MJ/m^2^)** |
| --- | --- |
| Calluna (Calluna vulgaris) | 25 |
| FestucaRubra (Agrostis curtisii) | 25 |
| Helictotrichon (Helictotrichon cantabricum) | 25 |
| Phragmites | 25 |
| Polypodiopsida | 25 |
| Senecio (sp) | 25 |
| UlexGallii | 25 |
| Carex | 25 |
| Gramineae | 25 |
| UlexEuropaeus | 25 |

S3C Table Equations of radiation namespace.

| **id** | **time =>0** |
| --- | --- |
| rg2 | if (com==com2) {  rg  } else {  rg * 0.2 } |
| fr | if (rg2 > rgc_max) {  1  } else {  rg2rgc_max } |
| frth | if ((ft*fr*fh)>1) {  1  } else {  ft*fr*fh } |

Temperature


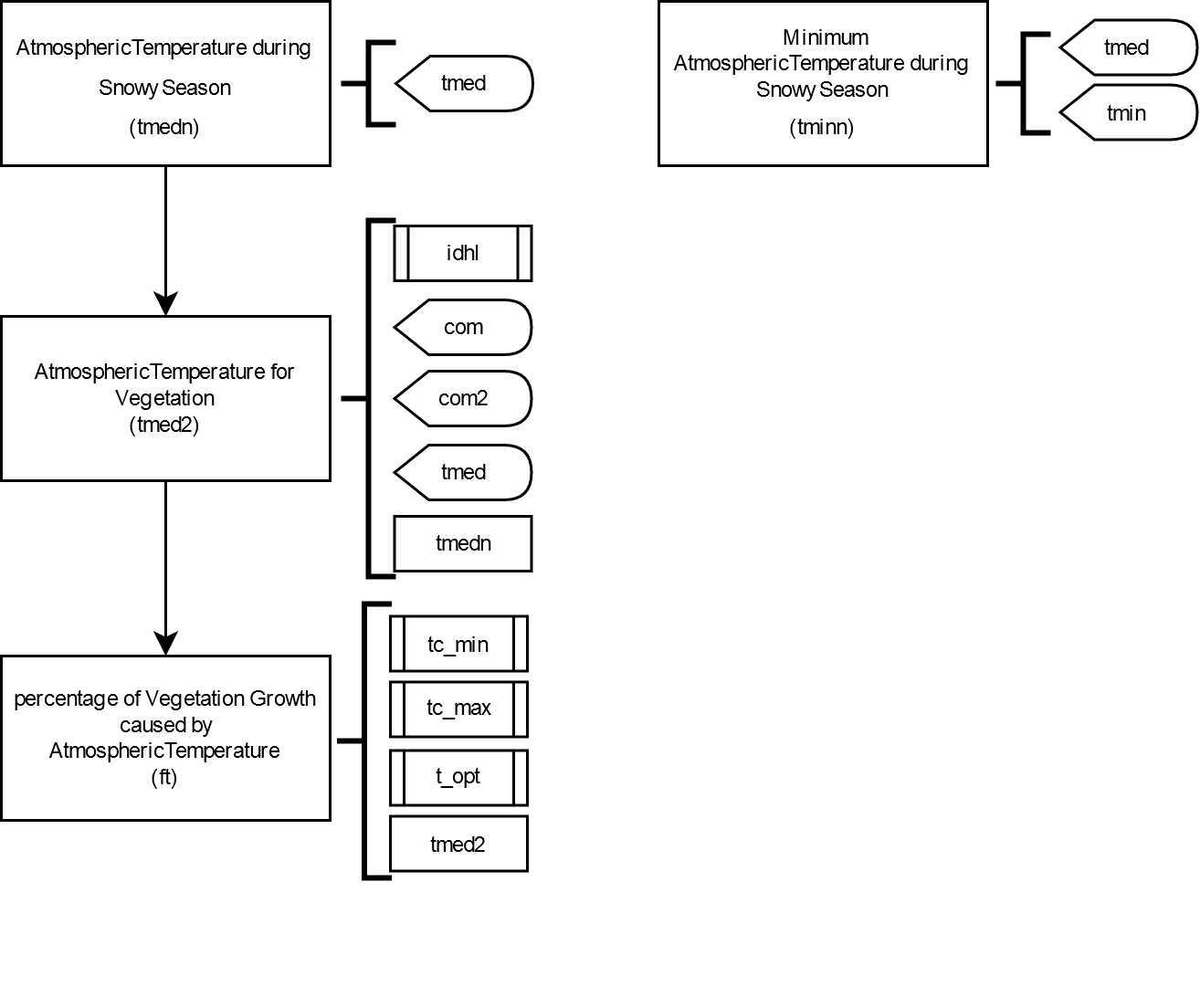


S4 Fig. Dataflow of temperature namespace.

S4A Table Description of temperature namespace models.

| **Puerto id** | **Semantic Model (k.IM language)** | **Description** | **Units** |
| --- | --- | --- | --- |
| ft | Percentage of Vegetation Growth caused by AtmosphericTemperature | Percentage of Atmospheric temperature that limits vegetation growth. 0: No growth (maximum limitation), 1: Maximum growth (no limitation) | [0-1] |
| idhl | Value of VegetationStratum | Vertical vegetation stratum coded as 1: ground layer; 2: shrubs layer; 3: midstory layer; 4: canopy layer | [1-4] |
| tc_max | Maximum AtmosphericTemperature causing Vegetation Growth | Maximum temperature limiting vegetation growth | °C |
| tc_min | Minimum AtmosphericTemperature causing Vegetation Growth | Minimum temperature limiting vegetation growth | °C |
| tmed | AtmosphericTemperature | Mean atmospheric temperature | °C |
| tmed2 | AtmosphericTemperature for Vegetation in Celsius | Vegetation temperature under woody plants | °C |
| tmedn | AtmosphericTemperature during Snowy Season | Mean atmospheric temperature over vegetation in snowy conditions | °C |
| tmin | Minimum AtmosphericTemperature | Minimum atmospheric temperature | °C |
| tminn | Minimum AtmosphericTemperature during Snowy Season in Celsius | Minimum atmospheric temperature over vegetation in snowy conditions | °C |
| topt | AtmosphericTemperature causing Maximum Vegetation Growth | Optimum temperature for vegetation growth | °C |

S4B Table Parameters of temperature namespace.

| **com** | **tc_min** | **tc_max)** | **topt** | **idhl** |
| --- | --- | --- | --- | --- |
| Calluna (Calluna vulgaris) | 4 | 35 | 21 | 2 |
| FestucaRubra (Agrostis curtisii) | 6 | 35 | 21 | 1 |
| Helictotrichon (Helictotrichon cantabricum) | 6 | 35 | 21 | 1 |
| Phragmites | 6 | 35 | 21 | 1 |
| Polypodiopsida | 9 | 35 | 21 | 3 |
| Senecio (sp) | 6 | 35 | 21 | 1 |
| UlexGallii | 6 | 35 | 21 | 2 |
| Carex | 5 | 35 | 21 | 1 |
| Gramineae | 6 | 35 | 21 | 1 |
| UlexEuropaeus | 9 | 35 | 21 | 2 |

S4C Table Equations of temperature namespace.

| **id** | **time =>0** |
| --- | --- |
| temdn | if (tmed<3) {  3  } else {  tmed } |
| tminn | if (tmed<3) {  3  } else {  tmin } |
| temd2 | if ((idhl==4)&&(com==com2)){  tmed  }else if (com2==com){  tmedn  }else{  (2+0.8*tmedn)} |
| fh | if (tmed2 < tc_min){  0  }if else (tmed2 > tc_max){  0  }else{  tc_max-tmed2tc_max-topt*tmed2-tc_mintopt-tc_mintopt-tc_mintmax-topt |

Vegetation growth


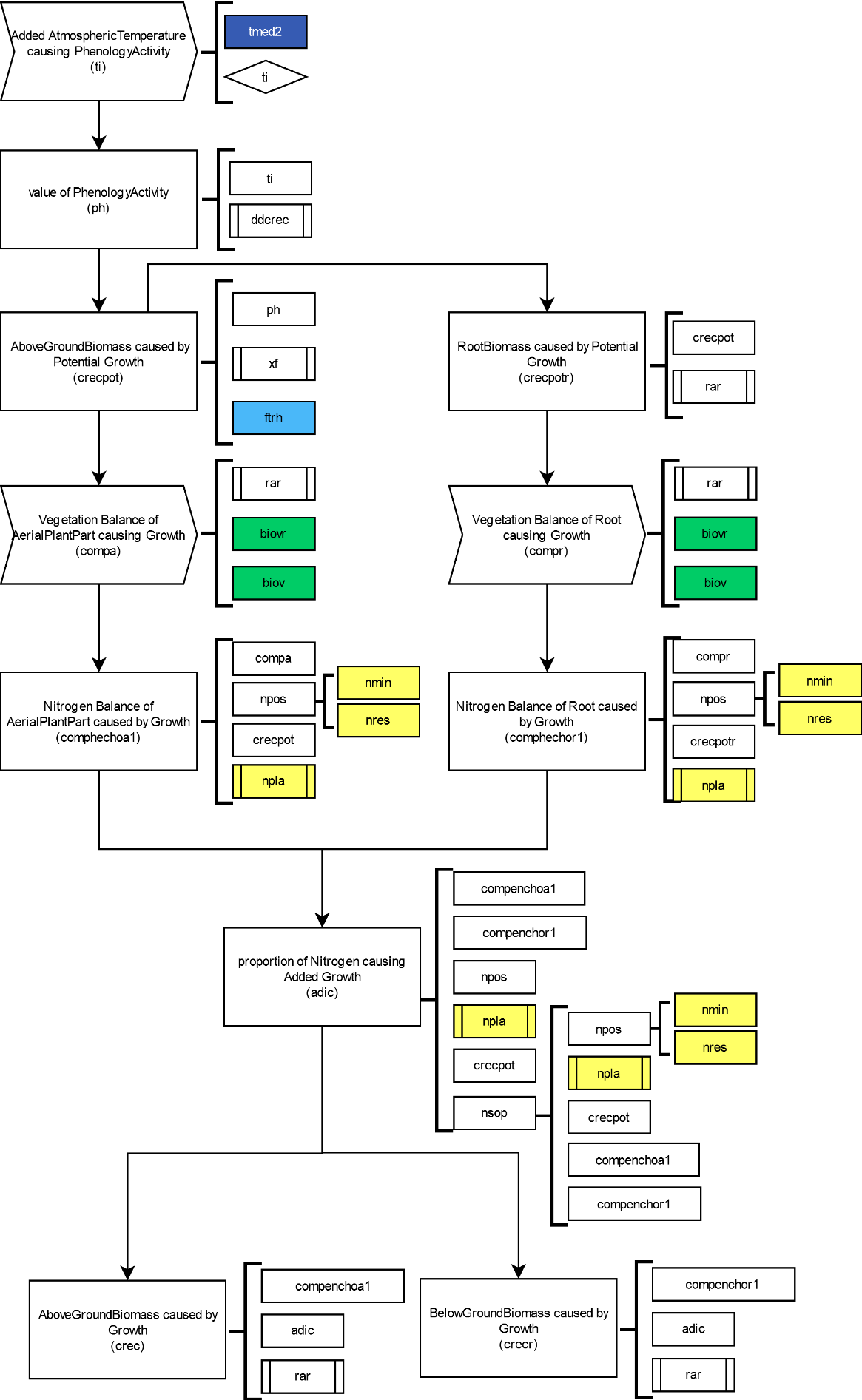


S5 Fig. Dataflow of vegetation growth namespace.

S5A Table Description of vegetation growth namespace models.

| **Puerto id** | **Semantic Model (k.IM language)** | **Description** | **Units** |
| --- | --- | --- | --- |
| adic | Proportion of Nitrogen causing Added Growth | Proportion of the remainder nitrogen causing extra vegetation growth | [0-1] |
| comp |  | Function to compute compa and compr |  |
| compa | Vegetation Balance of AerialPlantPart causing Growth | The remainder of net primary production is used for aerial vegetation growth. | g/m^2^ |
| comphechoa |  | Function to calculate comphechoa1 |  |
| comphechoa1 | Nitrogen Balance of AerialPlantPart caused by Growth | Normalised concentration of nitrogen in the aerial part of the plant | [0-1] |
| comphechor |  | Function to calculate comphechoar1 |  |
| comphechor1 | Nitrogen Balance of Root caused by Growth | Normalised concentration of nitrogen in the root part of the plant | [0-1] |
| compr | Vegetation Balance of Root causing Growth | The remainder of net primary production used for root growth | g/m^2^ |
| crec | AboveGroundBiomass caused by Growth | Actual growth of above ground biomass | g/m^2^ |
| crecpot | Potential AboveGroundBiomass caused by Potential Growth | Potential growth of above ground biomass | g/m^2^ |
| crecpotr | Potential RootBiomass caused by Potential Growth | Potential growth of below ground biomass | g/m^2^ |
| crecr | RootBiomass caused by Growth | Actual growth of below ground biomass | g/m^2^ |
| ddcrec | Added AtmosphericTemperature causing Active Growth in Celsius | Growing degree days (GDD) accumulated, a commonly used measure of thermal accumulation | °C |
| npos |  | Function that sums nres (nitrogen causing vegetation growth) and mnim (inorganic nitrogen) | g/m^2^ |
| ph | Occurrence of PhenologyActivity | Phenological state of vegetation | [0-1] |
| rar | Proportion of RootBiomass in Balance Vegetation | Relation between the growth of aerial and root system | [0-1] |
| ti | Added AtmosphericTemperature causing PhenologyActivity in Celsius | Accumulated temperature necessary to complete a phenological state | °C |
| xf | Maximum Biomass caused by Growth | Theoretical vegetation growth without limitations | g/m^2^ |

S5B Table Parameters of vegetation growth namespace.

| **com** | **xf** | **rar** | **ddcrec** |
| --- | --- | --- | --- |
| Calluna (Calluna vulgaris) | 3 | 0.5 | 10000 |
| FestucaRubra (Agrostis curtisii) | 10 | 0.35 | 10000 |
| Helictotrichon (Helictotrichon cantabricum) | 7 | 0.45 | 10000 |
| Phragmites | 6 | 0.35 | 10000 |
| Polypodiopsida | 5 | 0.35 | 2200 |
| Senecio (sp) | 4 | 0.35 | 10000 |
| UlexGallii | 5 | 0.35 | 10000 |
| Carex | 4 | 0.45 | 10000 |
| Gramineae | 8 | 0.35 | 10000 |
| UlexEuropaeus | 6 | 0.35 | 10000 |

S5C Table Equations of vegetation growth namespace.

| **id** | **time (t=>0)** | |
| --- | --- | --- |
|  | **t=0** | **t>0** |
| ti | if (tmed2 < 4) {  0  } else {  tmed2} | ti + tit-1 |
| ph | if (ti < ddcrec) {  1  } else {  0} | |
| crecpot | xf*ftrh*ph | |
| crecpotr | crecpot*rar1-rar | |
| comp | biovr-rar1-rar*biov | |
|  | nmin+nres | |
| compa | 0 | if (comp > 0) {  comp  } else {  0} |
| compr | 0 | if (comp< 0) {  -comp  } else {  0} |
| comphechoa | if npos > compa*npla{  compa  } else {  nposnpla} | |
| comphechoa1 | if comphechoa > crecpot{  crecpot  } else {  comphechoa} | |
| comphechor | if npos>(compr*npla) {  compr  } else {  nposnpla} | |
| comphechor1 | if comphechor > crecpotr{  crecpotr  } else {  comphechor} | |
| nsop | npos-comphechoa1*npla-comphechor1*npla | |
| adic | *a=*  crecpot-comphechoa1-comphechor1  if nsop > a*npla{  a  }else{  nsopnpla | |
| crec | comphechoa1 + adic*1-rar | |
| crecr | comphechor1 + adic*rar | |

Senescence


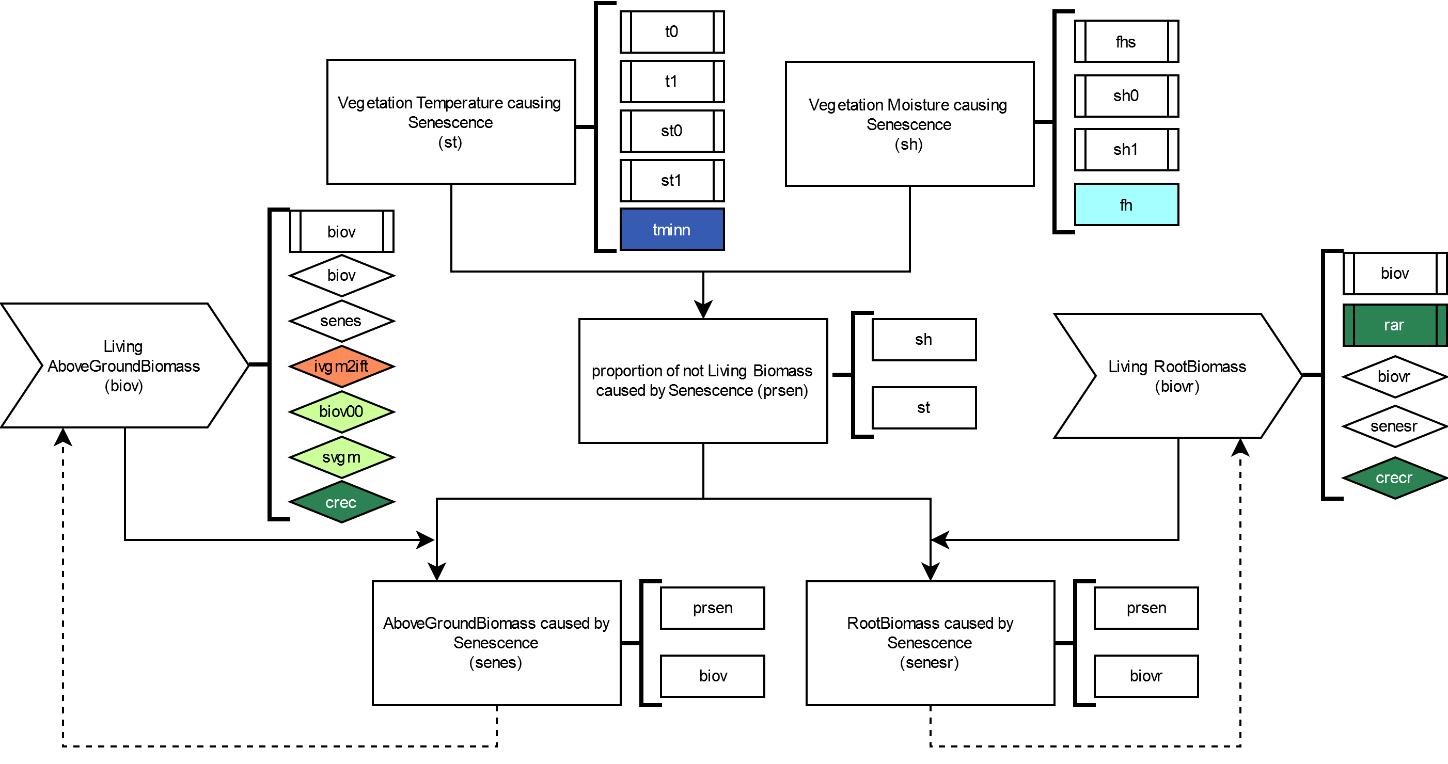


S6 Fig. Dataflow of the senescence namespace.

S6A Table Description of senescence namespace models.

| **Puerto id** | **Semantic Model (k.IM language)** | **Description** | **Unit** |
| --- | --- | --- | --- |
| biov | Living AboveGroundBiomass | Living above ground biomass | g/m^2^ |
| biovr | Living RootBiomass | Living below ground biomass | g/m^2^ |
| fhs | Proportion of Moisture causing Minimum Senescence | Level of moisture (fh) with minimum senescence | [0-1] |
| prsen | Proportion of not Living Biomass caused by Senescence | Maximum proportion of senescence caused by temperature or moisture | [0-1] |
| senes | AboveGroundBiomass caused by Senescence | Amount of above ground biomass dying caused by senescence | g/m^2^ |
| senesr | RootBiomass caused by Senescence | Amount of below ground biomass dying caused by senescence | g/m^2^ |
| sh | Vegetation Moisture causing Senescence | Proportion of leave senescence caused by moisture | [0-1] |
| sh0 |  | Maximum proportion of leaves dying due to moisture | [0-1] |
| sh1 |  | Proportion of minimum leave senescence caused by soil moisture | [0-1] |
| st | Vegetation AtmosphericTemperature causing Senescence | Proportion of senescence caused by temperature | [0-1] |
| st0 |  | Maximum proportion of leaves dying due to temperature | [0-1] |
| st1 |  | Proportion of temperature causing minimal senescence | [0-1] |
| t0 | AtmosphericTemperature causing Maximum Senescence | Atmospheric temperature producing maximum senescence | °C |
| t1 | AtmosphericTemperature causing Minimum Senescence | Atmospheric temperature producing minimum senescence | °C |

S6B Table Parameters of vegetation growth namespace.

| **com** | **fhs** | **sh0** | **sh1** | **t0** | **t1** | **st0** | **st1** | **biov** |
| --- | --- | --- | --- | --- | --- | --- | --- | --- |
| Calluna (Calluna vulgaris) | 0.3 | 0.005 | 0.002 | -5 | 3 | 0.5 | 0 | 100 |
| FestucaRubra (Agrostis curtisii) | 0.4 | 0.030 | 0.005 | -1 | 4 | 0.5 | 0 | 50 |
| Helictotrichon (Helictotrichon cantabricum) | 0.3 | 0.010 | 0.005 | -1 | 4 | 0.5 | 0 | 40 |
| Phragmites | 0.4 | 0.007 | 0.002 | -1 | 4 | 0.5 | 0 | 90 |
| Polypodiopsida | 0.3 | 0.010 | 0.005 | 5 | 9 | 0.5 | 0 | 0.1 |
| Senecio (sp) | 0.3 | 0.010 | 0.005 | -1 | 4 | 0.5 | 0 | 10 |
| UlexGallii | 0.4 | 0.010 | 0.002 | -1 | 4 | 0.5 | 0 | 50 |
| Carex | 0.3 | 0.003 | 0.001 | -8 | 4 | 0.5 | 0 | 10 |
| Gramineae | 0.4 | 0.030 | 0.008 | -1 | 4 | 0.5 | 0 | 80 |
| UlexEuropaeus | 0.4 | 0.010 | 0.002 | 0 | 8 | 0.5 | 0 | 100 |

S6C Table Equations of vegetation growth namespace.

| **id** | **time (t=>0)** | |
| --- | --- | --- |
|  | **t=0** | **t>0** |
| sh | if (fh < 0){  sh0  }if else (fh > fhs){  sh1  }else{  sh0 * fhs - fh- sh1*0 - fhfhs - 0 | |
| st | if (tminn < t0){  st0  }if else (tminn > t1){  st1  }else{  st0 * t1 - tminn- st1*t0 - tminnt1 - t0 | |
| prsen | Maximum [sh,st] | |
| biov | biov | *harvest =*  if (biov00 == null){  biov  }if else (biov > biov00){  biov00  }else{  biov  *hbiov =*  harvest+crec-senes-ivgm2ift-svgm    *biov =*  if hbiov < 0{  0  } else {  hbiov} |
| biovr | biov*rar1-rar | biovr+crecr-senesr |
| senes | prsen*biov | |
| senesr | prsen*biovr | |

Litterfall


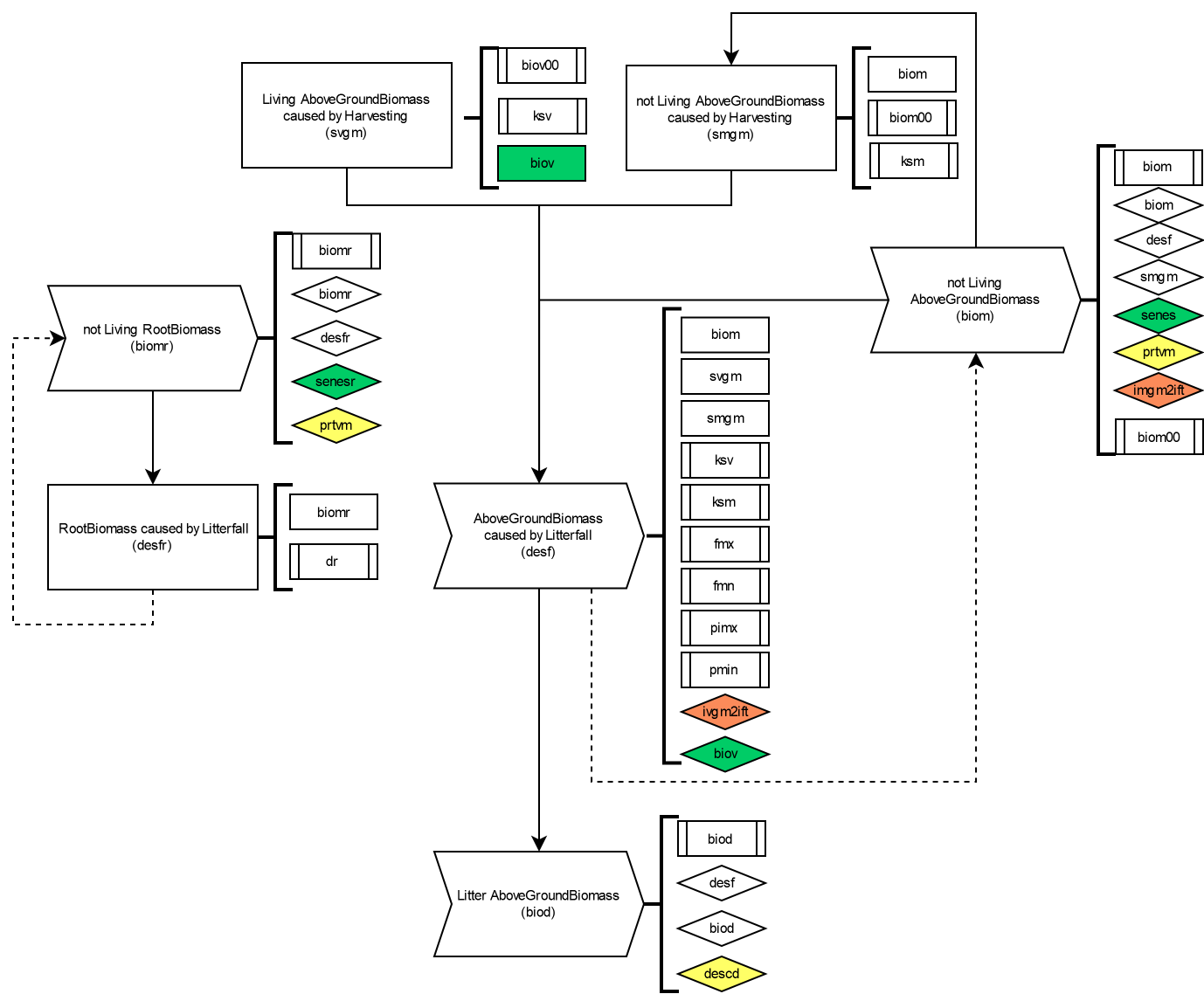


S7 Fig. Dataflow of litterfall namespace.

S7A Table Description of litterfall namespace models.

| **Puerto id** | **Semantic Model (k.IM language)** | **Description** | **Unit** |
| --- | --- | --- | --- |
| biod | Litter AboveGroundBiomass | Amount of litterfall related to above ground biomass | g/m^2^ |
| biom | Not Living AboveGroundBiomass | Dead standing above ground biomass | g/m^2^ |
| biom00 |  | Standing dead biomass remaining after harvest | g/m^2^ |
| biomr | Not Living RootBiomass | Dead standing root biomass | g/m^2^ |
| biov00 |  | Living standing biomass remaining after harvest | g/m^2^ |
| desf | AboveGroundBiomass caused by Litterfall | Process of litterfall related to above ground biomass | g/m^2^ |
| desfr | RootBiomass caused by Litterfall | Process of litterfall related to root biomass | g/m^2^ |
| dr |  | Rate of root litterfall |  |
| fmn | Proportion of Minimum Litterfall in not Living AboveGroundBiomass | Minimum proportion of litterfall caused by livestock | day |
| fmx | Proportion of Maximum Litterfall in not Living AboveGroundBiomass | Maximum proportion of litterfall caused by livestock | day |
| ksm | Proportion of not Living AboveGroundBiomass in Harvesting | Harvesting efficiency of dead above ground biomass | [0-1] |
| ksv | Proportion of Living AboveGroundBiomass in Harvesting | Harvesting efficiency of living above ground biomass | [0-1] |
| pimn | Proportion of AboveGroundBiomass in Minimum Litterfall | Proportion of ingested biomass by livestock with minimum fall | day |
| pimx | Proportion of AboveGroundBiomass in Maximum Litterfall | Proportion of ingested biomass by livestock with maximum fall | day |
| smgm | Not Living AboveGroundBiomass caused by Harvesting | Amount of dead biomass harvested | g/m^2^ |
| svgm | Living AboveGroundBiomass caused by Harvesting | Amount of living biomass harvested | g/m^2^ |

S7B Table Parameters of litterfall namespace.

| com | biov00 | biom00 | dr | ksv | ksm | pimx | pimn | biod | biom | biomr | fmx | fmn |
| --- | --- | --- | --- | --- | --- | --- | --- | --- | --- | --- | --- | --- |
| Calluna (Calluna vulgaris) | 5 | 10 | 0.2 | 0.9 | 0.8 | 0.05 | 0 | 80 | 40 | 40 | 0.01 | 0.002 |
| FestucaRubra (Agrostis curtisii) | 5 | 10 | 0.2 | 0.9 | 0.8 | 0.05 | 0 | 20 | 20 | 20 | 0.02 | 0.002 |
| Helictotrichon (Helictotrichon cantabricum) | 5 | 10 | 0.2 | 0.9 | 0.8 | 0.05 | 0 | 40 | 110 | 110 | 0.02 | 0.002 |
| Phragmites | 5 | 10 | 0.2 | 0.9 | 0.8 | 0.05 | 0 | 10 | 10 | 10 | 0.02 | 0.002 |
| Polypodiopsida | 5 | 10 | 0.2 | 0.9 | 0.8 | 0.05 | 0 | 80 | 200 | 200 | 0.02 | 0.002 |
| Senecio (sp) | 5 | 10 | 0.2 | 0.9 | 0.8 | 0.05 | 0 | 20 | 40 | 40 | 0.02 | 0.002 |
| UlexGallii | 5 | 10 | 0.2 | 0.9 | 0.8 | 0.05 | 0 | 50 | 100 | 100 | 0.01 | 0.002 |
| Carex | 5 | 10 | 0.2 | 0.9 | 0.8 | 0.05 | 0 | 10 | 10 | 10 | 0.02 | 0.002 |
| Gramineae | 5 | 10 | 0.2 | 0.9 | 0.8 | 0.05 | 0 | 20 | 20 | 20 | 0.02 | 0.002 |
| UlexEuropaeus | 5 | 10 | 0.2 | 0.9 | 0.8 | 0.05 | 0 | 100 | 200 | 200 | 0.01 | 0.002 |

S7C Table Equations of litterfall namespace.

| id | time (t=>0) | |
| --- | --- | --- |
|  | **t=0** | **t>0** |
| biod | biod | biod + desf - descd |
| biom | biom | *harvest =*  if (biom00 == null){  biom  }if else (biom > biom00){  biom00  }else{  biom  hbiom =harvest+senes*1-prtvm-desf-imgm2ift-smgm    *biov =*  if hbiom < 0{  0  } else {  hbiom} |
| biomr | biom | biomr+senesr*1-prtvm-desfr |
| desf | *dt =*  if (0<pimn){  fmn  }if else(0>pimx) {  fmx  }else{  fmn + fmx-fmnpimx-pimn*0-pimn}  *desf*=  dt*biom+ 1-ksv*svgm+ 1-ksm*smgm | *pi=*  if biov==0{  0  } else {  ivgm2iftbiov}  *dt_2* =  if (pi<pimn){  fmn  }if else(pi>pimx) {  fmx  }else{  fmn+fmx-fmnpimx-pimn*pi-pimn}   desf =  (dt*biom) + 1-ksv*svgm+ 1-ksm*smgm |
| desfr | biomr * dr | |
| smgm | if (biom00 == null){  0  }if else((ksm * biom) > biom00) {  ksm * biom- biom00  }else{  0} | |
| svgm | if (biov00 == null){  0  }if else((ksv * biov) > biov00) {  ksv * biov- biov00  }else{  0} | |

Livestock Ingestion


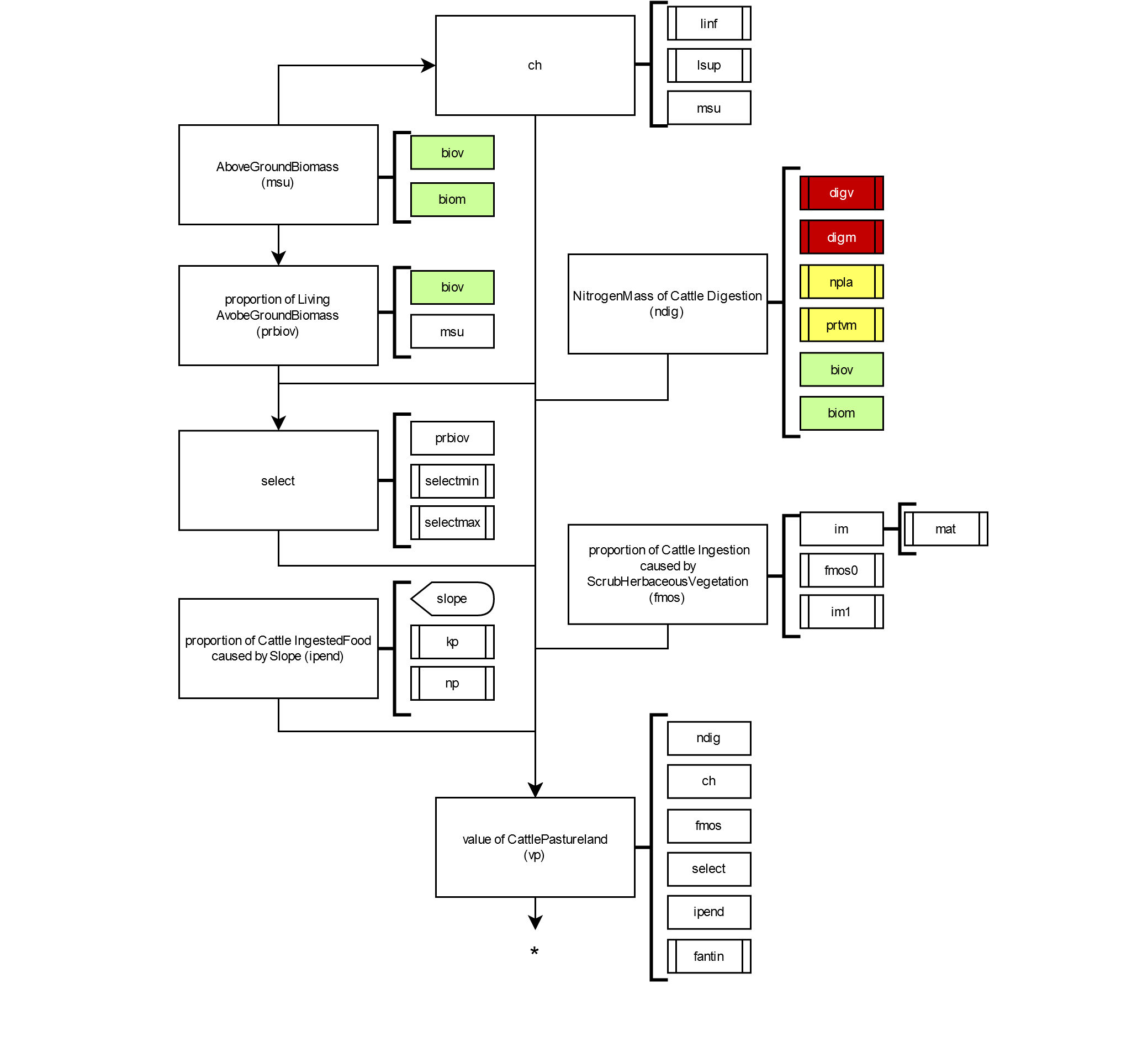


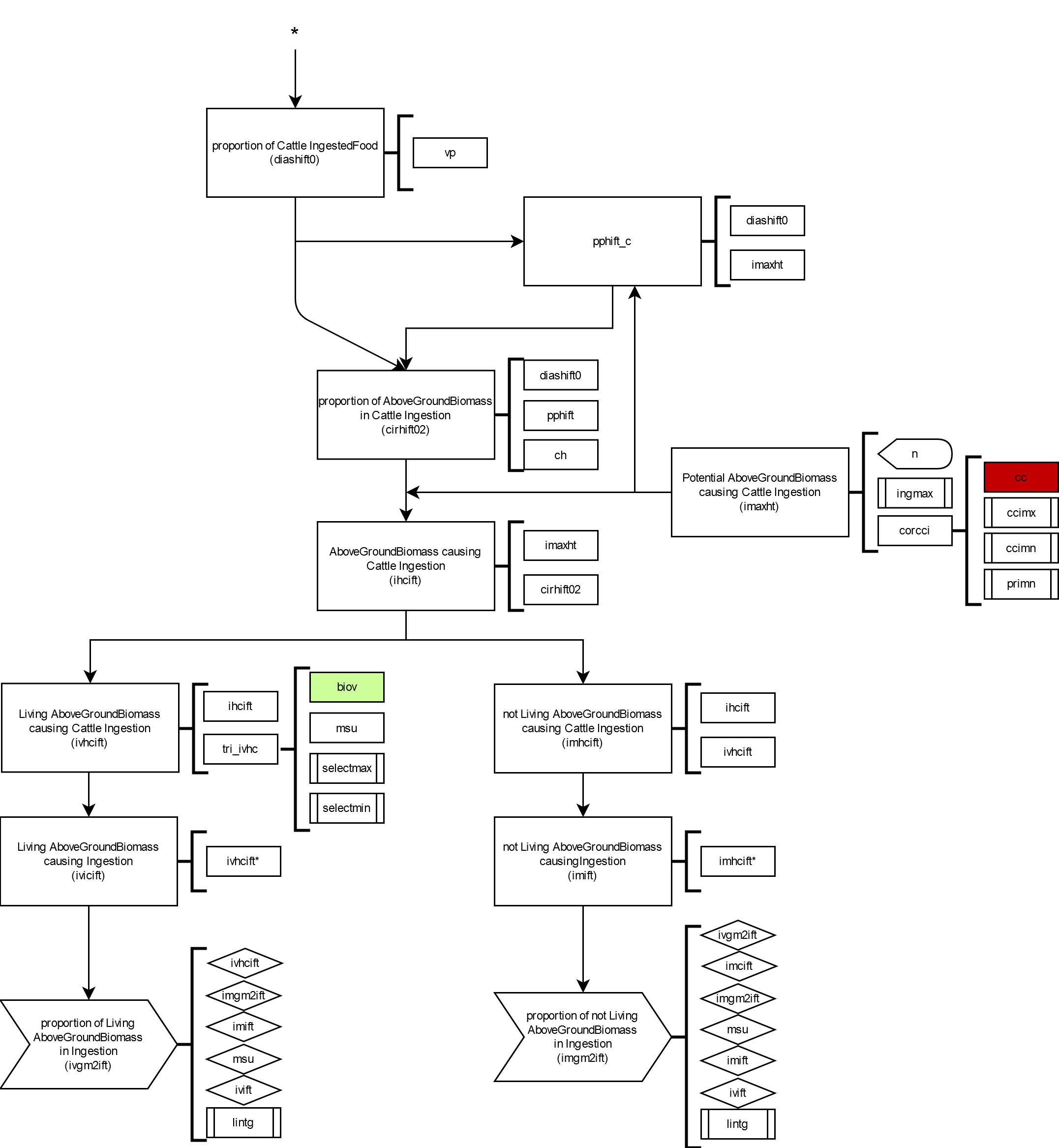


S8 Fig. Dataflow of ingestion namespace.

S8A Table Description of ingestion namespace models.

| **Puerto id** | **Semantic Model (k.IM language)** | **Description** | **Units** |
| --- | --- | --- | --- |
| ccimn |  | Body condition at minimal potential intake | [0-5] |
| ccimx |  | Body condition at maximum potential intake | [0-5] |
| ch |  | Coefficient (01) of regulation of livestock intake according to available biomass | [0-1] |
| cirhift01 |  | Potential proportion of total intake corresponding to type of grass | [0-1] |
| cirhift02 | Proportion of AboveGroundBiomass in Cattle Ingestion | Real proportion of the herd's ingestion corresponding to type of grass | [0-1] |
| corcci |  | Function to calculate the livestock body condition |  |
| diashift0 | Proportion of Cattle IngestedFood | Estimates the proportion of grazing if there were no overlap with others’ herds | [0-1] |
| fantin |  | Maximum proportion of (01) a type of grass can be part of the animal's daily diet | [0-1] |
| fmos | Proportion of Cattle Ingestion caused by ScrubHerbaceousVegetation | Difficulty of livestock access due to excess shrub growth (0: not accessible; 1 : fully accessible) | [0-1] |
| fmos0 |  | Minimum accessibility of livestock | [0-1] |
| ihcift | AboveGroundBiomass causing Cattle Ingestion | Amount of above ground biomass ingested by each type of livestock | kg/ha |
| im | Proportion of ScrubHerbaceousVegetation causing Cattle Movement | Livestock accessibility due to the shrubs proportion(0: no shrubs, 1: maximum proportion of shrubs) | [0-1] |
| im0 |  | Proportion of shrub intake with minimal livestock accessibility | [0-1] |
| im1 |  | Proportion of shrub intake with maximal livestock accessibility | [0-1] |
| imaxht | Potential AboveGroundBiomass causing Cattle Ingestion | Potential ingestion without biomass limitation | kg/ha |
| imgm2ift | Proportion of not Living AboveGroundBiomass in Ingestion | Proportion of ingested nonliving biomass in the whole of the ingested biomass | [0-1] |
| imhcift | Not Living AboveGroundBiomass causing Livestock  Ingestion | Nonliving above ground biomass ingested by each type of livestock | g |
| imift | Not Living AboveGroundBiomass causing Ingestion | Nonliving above ground biomass ingested by livestock | g |
| ingmax |  | Maximum ingestion | kg |
| ipend | Proportion of Cattle IngestedFood caused by Slope | Coefficient (01) of grazing capacity according to slope | [0-1] |
| ivgm2ift | Proportion of Living AboveGroundBiomass in Ingestion | Proportion of ingested living biomass out of all ingested biomass | [0-1] |
| ivhcift | Living AboveGroundBiomass causing Cattle Ingestion named ivhcift | Living above ground biomass ingested by each type of livestock | g |
| ivift | Living AboveGroundBiomass causing Ingestion | Living above ground biomass ingested by livestock | g |
| kp |  | Parameter that indicates the slope when the function of the model "proportion of Cattle IngestedFood caused by Slope" is 0.5 | % |
| linf |  | Amount of aerial biomass that prevents grazing | g/m^2^ |
| lintg |  | Amount of biomass below which there is no intake | g/m^2^ |
| lsup |  | Amount of aerial biomass that allows grazing | g/m^2^ |
| mat |  | Probability of accessibility and livestock movement through vegetation | [0-1] |
| msu | AboveGroundBiomass | Standing above ground biomass (living and nonliving) | g/m^2^ |
| ndig | Nitrogen Mass of Cattle Digestion | Digestible nitrogen mass of standing vegetation | g/m^2^ |
| np |  | Function parameter indicating the decrease in the "proportion of feed intake by livestock caused by the slope" model when the slope increases |  |
| pphift |  | Proportion of potential grazing of a given pasture corresponding to a herd | [0-1] |
| prbiov | Proportion of Living AvobeGroundBiomass | Proportion of living above ground biomass | [0-1] |
| primn |  | Proportion of potential intake to “ccimn” model | [0-1] |
| select |  | Coefficient (01) indicating the livestock ability to select the living against the dead part of the vegetation | [0-1] |
| selectmax | Maximum proportion of Living AboveGroundBiomass in IngestedFood | Proportion of living biomass that allows the livestock to select only living vegetation | [0-1] |
| selectmin | Minimum proportion of Living AboveGroundBiomass in IngestedFood | Proportion of living biomass that prevents the livestock from selecting living vegetation | [0-1] |
| slope | Slope | Terrain inclination | % |
| tri_ivhc |  | Function to calculate ingestion model |  |
| vp | Value of Cattle Gramineae | Gramineae quality according to its composition | g/m^2^ |

S8B Table Parameters of ingestion namespace.

| **com** | **selectmax** | **selectmin** | **kp** | **np** | **lintg** | **ccimx** | **ccmin** | **primn** | **im1** | **fmos0** | **fantin (cattle)** | **mat (cattle)** | **fantin (mares)** | **mat (mares)** | **linf** | **lsup** | **ingmax** |
| --- | --- | --- | --- | --- | --- | --- | --- | --- | --- | --- | --- | --- | --- | --- | --- | --- | --- |
| Calluna (Calluna vulgaris) | 0.9 | 0.2 | 25 | 5 | 26.8 | 2.5 | 3.5 | 0.5 | 0.6 | 0 | 0.2 | 0 | 0 | 0 |  |  |  |
| FestucaRubra (Agrostis curtisii) | 0.9 | 0.2 | 25 | 5 | 26.8 | 2.5 | 3.5 | 0.5 | 0.6 | 0 | 1 | 0 | 1 | 0 |  |  |  |
| Helictotrichon (Helictotrichon cantabricum) | 0.9 | 0.2 | 25 | 5 | 26.8 | 2.5 | 3.5 | 0.5 | 0.6 | 0 | 1 | 0 | 1 | 0 |  |  |  |
| Phragmites | 0.9 | 0.2 | 25 | 5 | 26.8 | 2.5 | 3.5 | 0.5 | 0.6 | 0 | 1 | 0.9 | 1 | 0.9 |  |  |  |
| Polypodiopsida | 0.9 | 0.2 | 25 | 5 | 26.8 | 2.5 | 3.5 | 0.5 | 0.6 | 0 | 0.3 | 0 | 1 | 0 |  |  |  |
| Senecio (sp) | 0.9 | 0.2 | 25 | 5 | 26.8 | 2.5 | 3.5 | 0.5 | 0.6 | 0 | 0.01 | 0 | 0.01 | 0 |  |  |  |
| UlexGallii | 0.9 | 0.2 | 25 | 5 | 26.8 | 2.5 | 3.5 | 0.5 | 0.6 | 0 | 0 | 0 | 0 | 0 |  |  |  |
| Carex | 0.9 | 0.2 | 25 | 5 | 26.8 | 2.5 | 3.5 | 0.5 | 0.6 | 0 | 0.05 | 1 | 0.2 | 1 |  |  |  |
| Gramineae | 0.9 | 0.2 | 25 | 5 | 26.8 | 2.5 | 3.5 | 0.5 | 0.6 | 0 | 0.05 | 1 | 0.1 | 1 |  |  |  |
| UlexEuropaeus | 0.9 | 0.2 | 25 | 5 | 26.8 | 2.5 | 3.5 | 0.5 | 0.6 | 0 | 1 | 0 | 1 | 0 |  |  |  |
| Cattle |  |  |  |  |  |  |  |  |  |  |  |  |  |  | 50 | 120 | 13.55 |
| Mares |  |  |  |  |  |  |  |  |  |  |  |  |  |  | 30 | 90 | 15.25 |

S8C Table Equations of ingestion namespace.

| id | time (t=>0) | |
| --- | --- | --- |
|  | **t=0** | **t>0** |
| ch | if (msu < linf){  0  }if else(msu > lsup) {  1  }else{  -1*linf - msulsup - linf} | |
| cirhift01 | diashift0*pphift | |
| cirhift02 | if (cirhift01 <= ch_c){  cirhift01  }else{  ch} | |
| corcci | if (cc < ccimx){  1  }if else (cc > ccimn) {  0.5  }else{  1+ 1-primnccimx-ccimn* cc-ccimx} | |
| diashift0 | [(vp == 0) ? 0 : 1] | |
| fmos | if (im < fmos0){  fmos0  }if else(im > im1) {  1  }else{  imim1} | |
| ihcift | imaxhct*cirhift02 | |
| im | 1 - mat | |
| imaxhct | n*ingmax*corcci | |
| imaxht | n*ingmax*corcci | |
| imgm2ift | 0 | *primift=*  if ((ivift+imift)==0){  0  }else{  imiftivift - imift}    *imgm2ift=*  if (ivgm2ift+imgm2ift > 0){  if(msu - ivhcift -ivgm2ift-imgm2ift) < lintg) {  (msu - lintg) * primift  }else{  imgm2ift  }else{  0} |
| imhcift | ihcift – ivhcift | |
| imift | imhcift_c + imhcift_m | |
| ipend | 1-slopenpslopenp+kpnp | |
| ivgm2ift | 0 | *privift=*  if ((ivift+imift)==0){  0  }else{  iviftivift - imift}    *ivgm2ift***=**  if (ivgm2ift+imgm2ift> 0){  if(msu - ivgm2ift-imgm2ift) < lintg) {  (msu - lintg) * privift  }else{  ivgm2ift  }else{  0} |
| ivhcift | ihcift*select | |
| ivift | ivhcift_c + ivhcift_m | |
| msu | biov+biom | |
| ndig | (digv *npla*biov) + (digm *npla*prtvm*biom) | |
| ppnum | imaxht*diashift0 | |
| prbiov | if (msu == 0){  0  }else{  biovmsu} | |
| select | if (prbiov < selectmin){  0  }if else(prbiov > selectmax) {  1  }else{  -1*selectmin-prbiovselectmax-selectmin} | |
| tri_ivhc | if biovmsu<selectmin{  0  }if elsebiovmsu<selectmax {  1  }else{  -1*selectmin-biovmsuselectmax-selectmin} | |
| vp | ndig * ch * fantin * fmos * ipend * tri_vp | |

Livestock Excretion


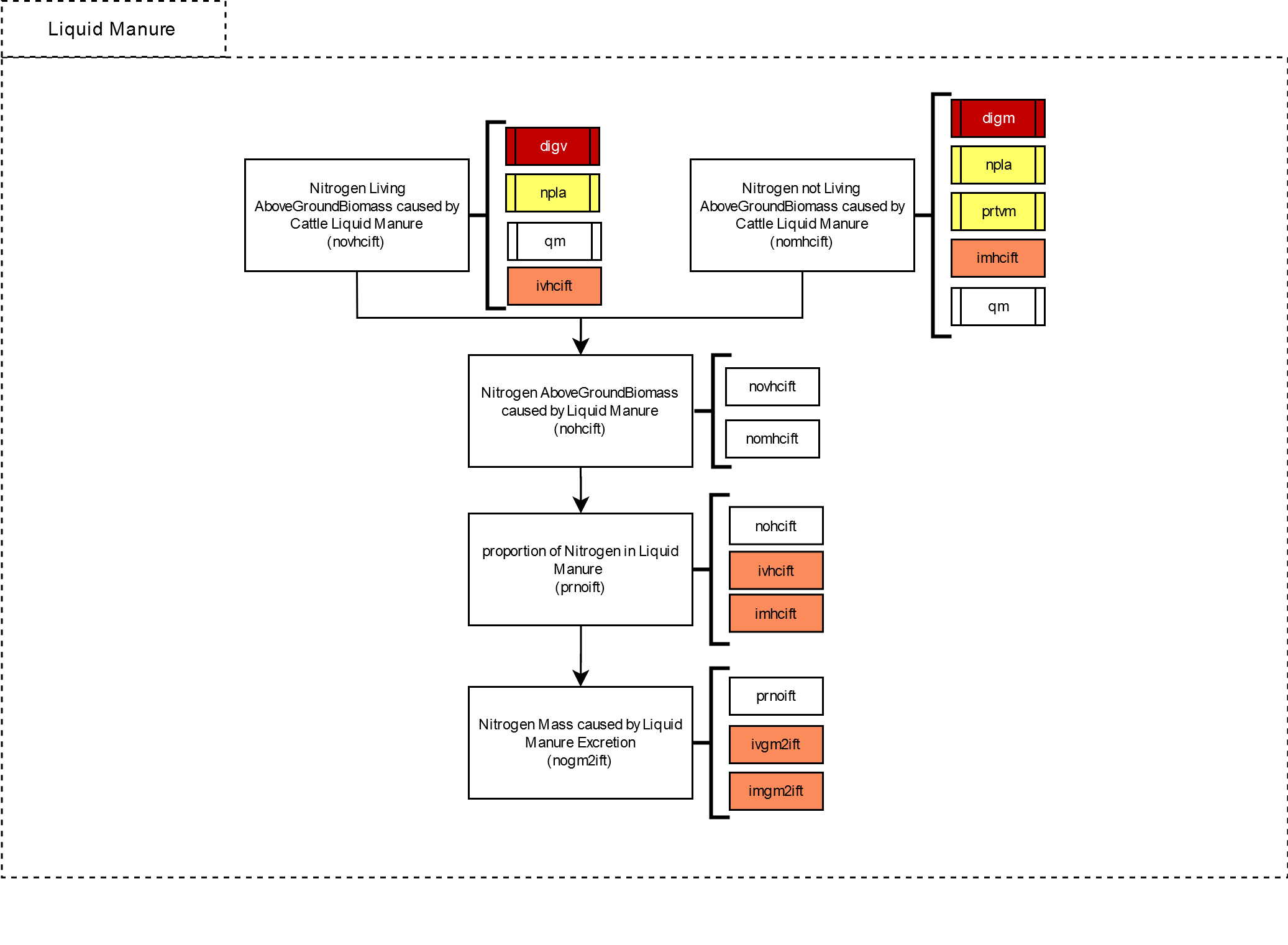


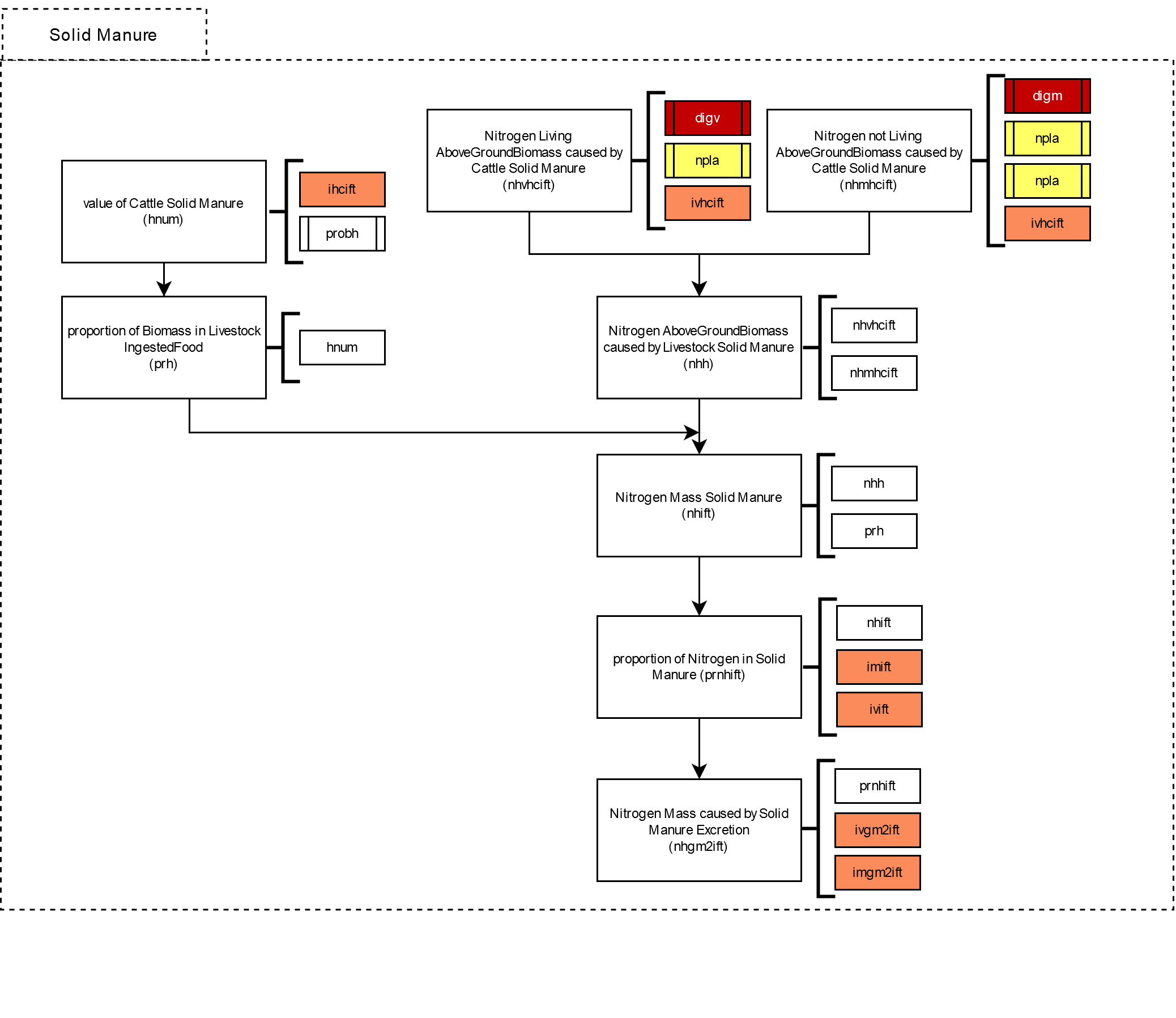


S9 Fig. Dataflow of excretion namespace.

S9A Table Description of excretion namespace models.

| Puerto id | Semantic Model (k.IM language) | Description | Units |
| --- | --- | --- | --- |
| qm |  | Coefficient of energy metabolism | [0-1] |
| nhmhcift | Nitrogen not  Living  AboveGroundBiomass caused by  Cattle Solid  Manure | Nitrogen content of nonliving  AboveGroundBiomass caused by Cattle Solid Manure | g/m^2^ |
| hnum | Cattle Solid  Manure | Livestock Solid Manure | g/m^2^ |
| nhgm2ift | Nitrogen  Mass caused by Solid  Manure  Excretion | Spatial distribution of solid manure nitrogen concentration | g/m^2^ |
| nhhift | Nitrogen  AboveGroundBiomass caused by  Livestock Solid  Manure | Nitrogen AboveGroundBiomass caused by Livestock Solid Manure | g/m^2^ |
| nhift | Nitrogen  Mass Solid  Manure | Nitrogen concentration of solid manure | g/m^2^ |
| nhvhcift | Nitrogen  Living  AboveGroundBiomass caused by  Cattle Solid  Manure | Nitrogen mass of living biomass in manure | g/m^2^ |
| nogm2ift | Nitrogen  Mass caused by Liquid  Manure  Excretion | Concentration of nitrogen in liquid manure | g/m^2^ |
| nohcift | Nitrogen  AboveGroundBiomass caused by Liquid  Manure | Nitrogen concentration of liquid manure caused by total of aboveground biomass ingestion | g/m^2^ |
| nomhcift | Nitrogen not Living AboveGroundBiomass caused by Cattle Liquid Manure | Nitrogen concentration of liquid manure caused by total of non living aboveground biomass ingestion | g/m^2^ |
| norinmhif | Nitrogen not  Living  AboveGroundBiomass caused by  Cattle Liquid  Manure | Nitrogen concentration of liquid manure caused by non living aboveground biomass ingestion | g/m^2^ |
| novhcift | Nitrogen  Living  AboveGroundBiomass caused by  Cattle Liquid  Manure | Nitrogen concentration of liquid manure caused by aboveground biomass ingestion | g/m^2^ |
| prh | Proportion of  Biomass in  Livestock  IngestedFood | proportion of Biomass in Livestock IngestedFood | [0-1] |
| prnhift | Proportion of Nitrogen in Solid  Manure | Proportion of nitrogen in manure | [0-1] |
| prnoift | Proportion of Nitrogen in Liquid  Manure | Proportion of nitrogen in liquid part of manure | [0-1] |
| probh | Occurrence of  Excretion | Probability of manure left in a plant community compared to its use | [0-1] |

S9B Table Parameters of excretion namespace.

| com | probh | qm |
| --- | --- | --- |
| Calluna (Calluna vulgaris) | 0.3 | 0.8 |
| FestucaRubra (Agrostis curtisii) | 0.8 | 0.8 |
| Helictotrichon (Helictotrichon cantabricum) | 0.2 | 0.8 |
| Phragmites | 0.6 | 0.8 |
| Polypodiopsida | 0.4 | 0.8 |
| Senecio (sp) | 0.8 | 0.8 |
| UlexGallii | 0.3 | 0.8 |
| Carex | 0.5 | 0.8 |
| Gramineae | 0.8 | 0.8 |
| UlexEuropaeus | 0.3 | 0.8 |

S9C Table Equations of excretion namespace.

| id | time (t=>0) |
| --- | --- |
| novhcift_c | ivhcift_c*digv*(1-qm)*npla |
| nomhcift_c | imhcift_c*digm_c*(1-qm)*npla*prtvm |
| nohcift | nohct = novhcif_c + novhcif_m + nomhcif_c + nomhcif_m  result = nohct*prh |
| prnoift | if ((ivift+imift)==0){  0  }else{  nohcift/(ivift+imift)} |
| nogm2ift | (ivgm2ift+imgm2ift)*prnoift |

Livestock mass


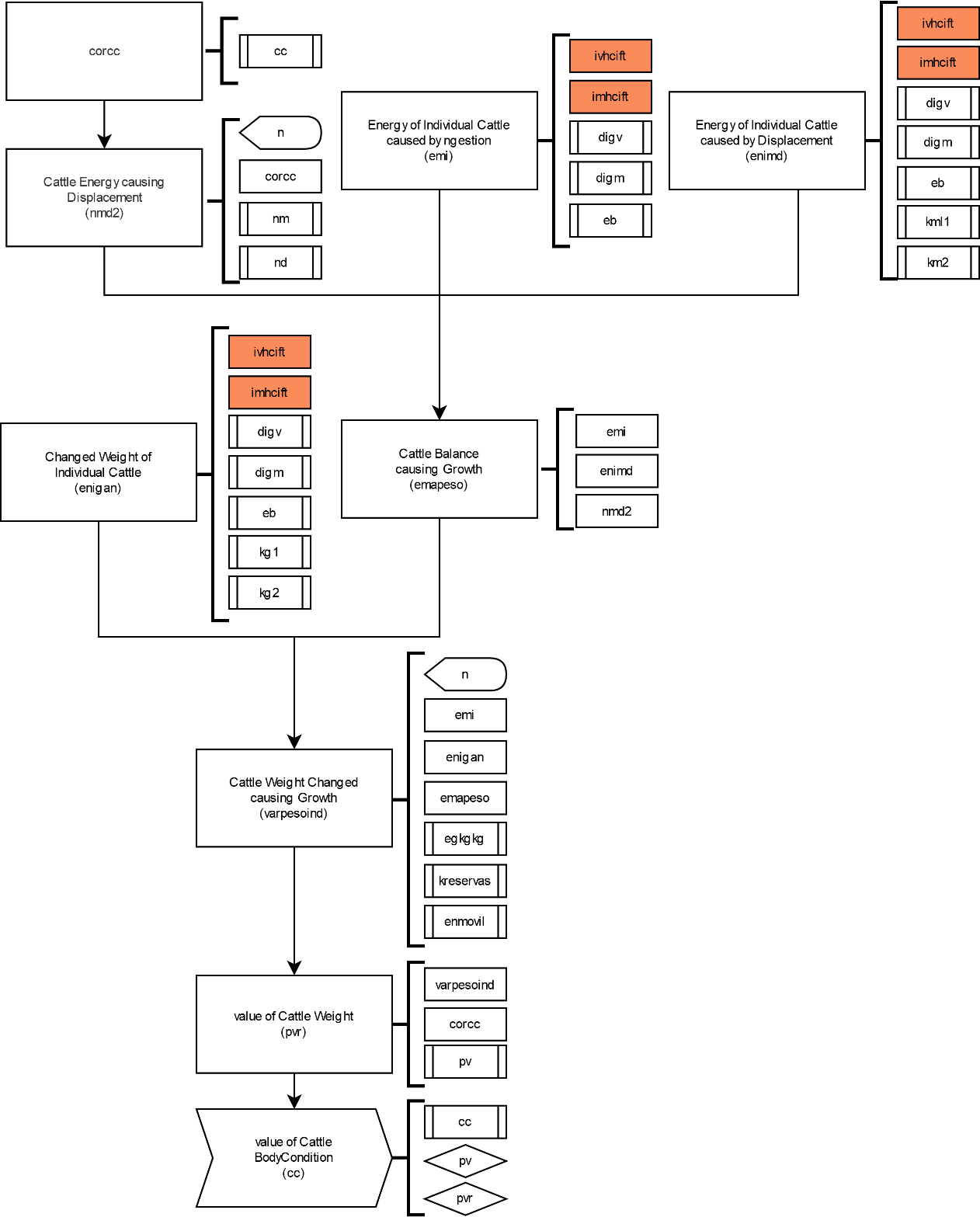


S10 Fig. Dataflow of livestock mass namespace.

S10A Table Description of livestock mass namespace models.

| Puerto id | Semantic Model (k.IM language) | Description | Units |
| --- | --- | --- | --- |
| cc | Value of Cattle BodyCondition | Body Condition | [0-5] |
| digm | Proportion of not Living AboveGroundBiomass in Cattle Digestion | Digestibility of nonliving aerial biomass | [0-1] |
| digv | Proportion of Living AboveGroundBiomass in Cattle Digestion | Digestibility of living aerial biomass | [0-1] |
| eb |  | Gross energy value | MJ/kg |
| egkg |  | Energy needed to increase 1 kg of body weight | MJ/kg |
| emapeso | Cattle Balance causing Growth | Balance between ingested energy and needs | MJ |
| emi | Energy of Individual Cattle caused by Ingestion | Metabolizable energy intake | MJ |
| enigan | Cattle Energy causing Changed Weight | Ingested energy that implies change of weight | MJ |
| enimd | Energy of Individual Cattle caused by Displacement | Ingested energy that implies movement and displacement | MJ |
| enmovil |  | Energy value of mobilizing body reserves | MJ/kg |
| kg1 |  | Energy efficiency to gain weight | [0-1] |
| kg2 |  | Metabolism energy efficiency for gain weight | [0-1] |
| km2 |  | Metabolism energy efficiency for maintenance and mobility | [0-1] |
| kml1 |  | Efficiency of energy use for lactation | [0-1] |
| kreservas |  | Efficiency of metabolizable energy used for food supplies mobilization | [0-1] |
| nmd2 | Cattle Energy causing Displacement | Livestock movement and displacement energy | MJ |
| pv | Reference Cattle Weight | Reference livestock weight | kg |
| pvr | Cattle Weight | Livestock weight | kg |
| varpesoind | Cattle Weight Changed causing Growth | Change of weight | g |

S10B Table Parameters of livestock mass namespace.

| com | digv_c | digv_m | digm | eb | kml1 | kreservas | enmovil | egkg | kg1 | pv | km2 | kg2 | nm | nd |
| --- | --- | --- | --- | --- | --- | --- | --- | --- | --- | --- | --- | --- | --- | --- |
| Calluna (Calluna vulgaris) | 0.5 | 0.45 | 0.15 |  |  |  |  |  |  |  |  |  |  |  |
| FestucaRubra (Agrostis curtisii) | 0.7 | 0.65 | 0.15 |  |  |  |  |  |  |  |  |  |  |  |
| Helictotrichon (Helictotrichon cantabricum) | 0.45 | 0.4 | 0.15 |  |  |  |  |  |  |  |  |  |  |  |
| Phragmites | 0.5 | 0.45 | 0.15 |  |  |  |  |  |  |  |  |  |  |  |
| Polypodiopsida | 0.55 | 0.5 | 0.15 |  |  |  |  |  |  |  |  |  |  |  |
| Senecio (sp) | 1 | 1 | 0.15 |  |  |  |  |  |  |  |  |  |  |  |
| UlexGallii | 0.4 | 0.35 | 0.15 |  |  |  |  |  |  |  |  |  |  |  |
| Carex | 0.7 | 0.65 | 0.15 |  |  |  |  |  |  |  |  |  |  |  |
| Gramineae | 0.7 | 0.65 | 0.15 |  |  |  |  |  |  |  |  |  |  |  |
| UlexEuropaeus | 0.4 | 0.35 | 0.15 |  |  |  |  |  |  |  |  |  |  |  |
| Cattle |  |  |  | 18.4 | 0.28 | 0.84 | 22.4 | 26 | 0.54 | 531.25 | 0.5 | 0.006 | 38.31 | 9.59 |
| Mares |  |  |  | 18.4 | 0.28 | 0.84 | 22.4 | 26 | 0.54 | 500 | 0.6 | 0.1 | 36.75 | 14.7 |

S10C Table Equations of livestock mass namespace.

| id | time (t=>0) | |
| --- | --- | --- |
|  | **t=0** | **t>0** |
| cc | 2.5 | 3+((pvr-pv)*5/pv) |
| corcc | 1+((cc_c-3)*0.2) | |
| nmd2 | (nm_c+nd_c)*corcc_c*n | |
| emi | (ivhcift_c*eb*digv_c*0.8)+(imhcift_c*eb*digm_c*0.8) | |
| enimd | ((ivhcift_c*eb*digv_c*0.8)*(kml1*digv_c+km2_c))+((imhcift_c*eb*digm_c*0.8)*(kml1*digm_c+km2_c)) | |
| enigan | ((ivhcift_c*eb*digv_c*0.8)*(kg1*digv_c+kg2_c))+((imhcift_c*eb*digm_c*0.8)*(kg1*digm_c+kg2_c)) | |
| emapeso | *kmprom* =  if (emi == 0){  1  }else{  enimd/emi  *result* =  emi-(nmd2/kmprom) | |
| varpesoind | *kganprom =*  if (emi == 0){  0  }else{  enigan/emi  *a =*  if emapeso < 0 {  emapeso*kreservas/enmovil  } else {  emapeso*kganprom/egkg  *result* =  a*1000/n | |
| pvr | pv + varpesoind | |

Nitrogen cycle


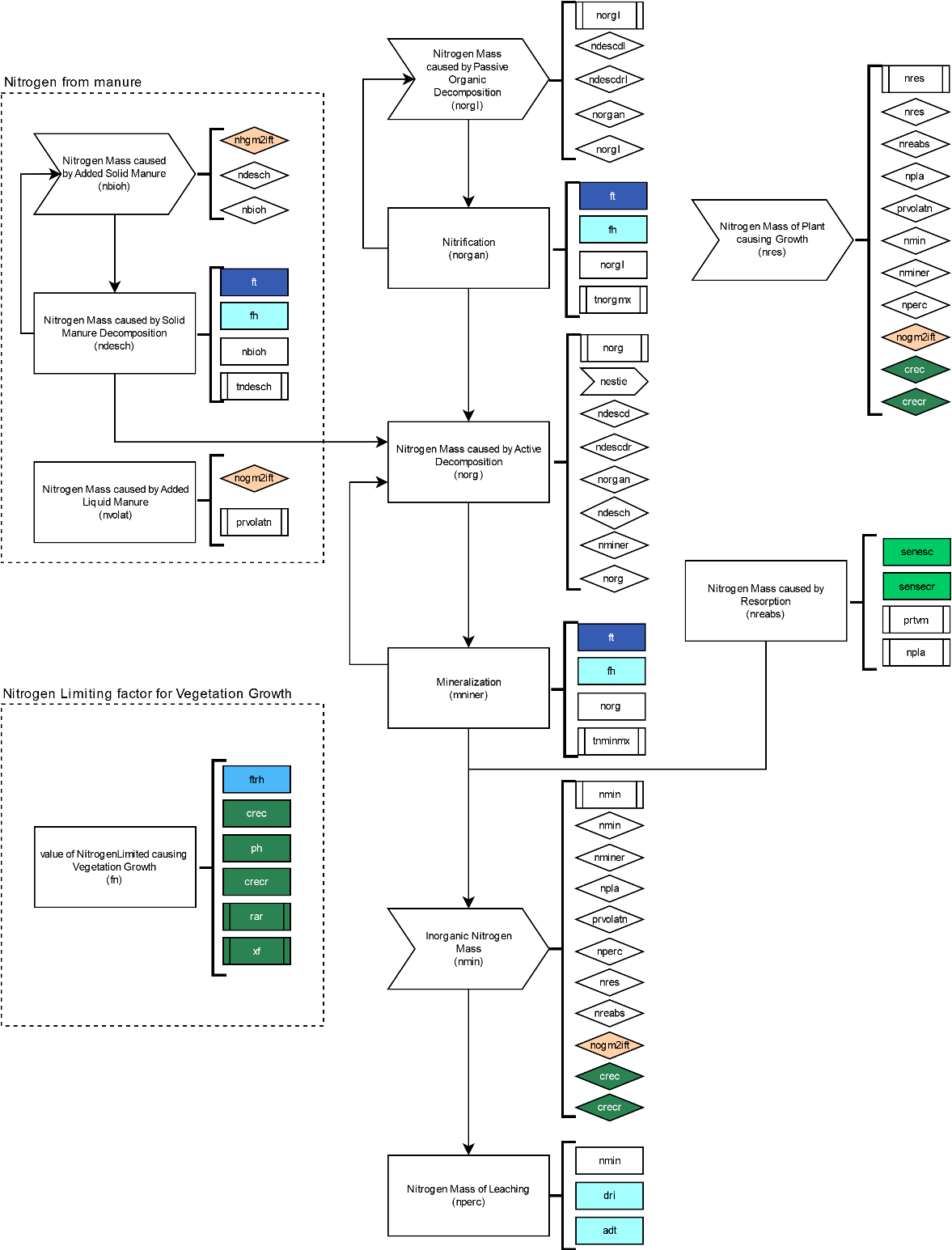


S11A Fig. Dataflow of nitrogen cycle namespace.


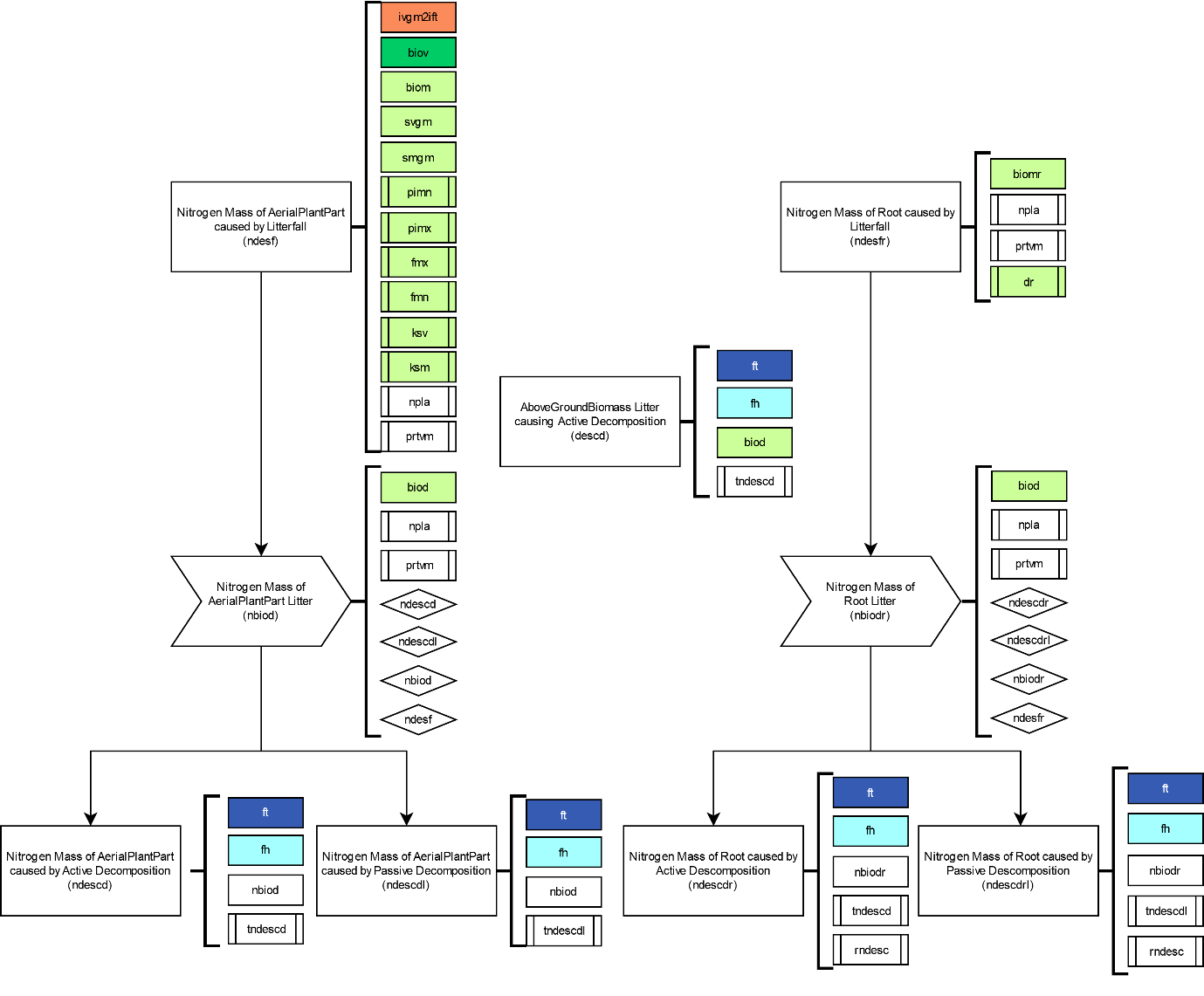


S11B Fig. Dataflow of nitrogen cycle namespace.

S11A Table Description of nitrogen cycle namespace models.

| **Puerto id** | **Semantic Model (k.IM language)** | **Description** | **Units** |
| --- | --- | --- | --- |
| descd | AboveGroundBiomass Litter causing Active Decomposition | Amount of litterfall of aerial vegetation biomass in active decomposition process | g/m^2^ |
| descdl | AboveGroundBiomass Litter causing Passive Decomposition | Amount of litterfall of aerial vegetation biomass in passive decomposition process | g/m^2^ |
| fn | Value of NitrogenLimited causing Vegetation Growth | Range of nitrogen mass that limits vegetation growth | [0-1] |
| nbiod | Nitrogen Mass of AerialPlantPart Litter | Amount of nitrogen in litterfall from aerial vegetation part | g/m^2^ |
| nbiodr | Nitrogen Mass of Root Litter | Amount of nitrogen in litterfall from root vegetation part | g/m^2^ |
| nbioh | Nitrogen Mass caused by Added Solid Manure | Amount of nitrogen accumulation from solid manure | g/m^2^ |
| ndescd | Nitrogen Mass of AerialPlantPart caused by Active Decomposition | Nitrogen mass in active decomposition (aerial vegetation part) | g/m^2^ |
| ndescdl | Nitrogen Mass of AerialPlantPart caused by Passive Decomposition | Nitrogen mass in passive decomposition (aerial vegetation part) | g/m^2^ |
| ndescdr | Nitrogen Mass of Root caused by Active Decomposition | Nitrogen mass in active decomposition (root vegetation part) | g/m^2^ |
| ndescdrl | Nitrogen Mass of Root caused by Passive Decomposition | Nitrogen mass in passive decomposition (root vegetation part) | g/m^2^ |
| ndesch | Nitrogen Mass caused by Solid Manure Decomposition | The amount of nitrogen that changes from solid manure decomposition to active organic nitrogen | g/m^2^ |
| ndesf | Nitrogen Mass of AerialPlantPart caused by Litterfall | Amount of nitrogen in aerial biomass litterfall | g/m^2^ |
| ndesfr | Nitrogen Mass of Root caused by Litterfall | Amount of nitrogen in root biomass litterfall | g/m^2^ |
| nestie | Nitrogen Mass causing Fertilization | Amount of organic nitrogen added by farmer as fertilizer | g/m^2^ |
| nmin | Inorganic Nitrogen Mass | Amount of inorganic nitrogen | g/m^2^ |
| nminer | Mineralization | Mineralization process | g/m^2^ |
| norg | Nitrogen Mass caused by Active Decomposition | Amount of nitrogen in active decomposition | g/m^2^ |
| norgan | Nitrification | Nitrification process |  |
| norgl | Nitrogen Mass caused by Passive Organic Decomposition | Amount of nitrogen in passive organic decomposition | g/m^2^ |
| nperc | Nitrogen Mass of Leaching | Amount of leaching nitrogen | g/m^2^ |
| npla | Proportion of Nitrogen in Living AboveGroundBiomass | Proportion of nitrogen in living above ground biomass |  |
| nreabs | Nitrogen Mass caused by Resorption | Amount of nitrogen in resorption process | g/m^2^ |
| nres | Nitrogen Mass of Plant causing Growth | Amount of nitrogen causing vegetation growth | g/m^2^ |
| nvolat | Nitrogen Mass caused by Added Liquid Manure | Amount of nitrogen accumulation from liquid manure | g/m^2^ |
| prtvm | Proportion of Nitrogen Mass in Resorption | Proportion of nitrogen in resorption process | [0-1] |
| prvolatn | Proportion of Nitrogen in Volatilization | Proportion of volatilized liquid manure | [0-1] |
| tndescd |  | Maximum proportion of litterfall nitrogen in active organic matter | [0-1] |
| tndescdl |  | Maximum proportion of litterfall nitrogen in passive organic matter | [0-1] |
| tndesch |  | Maximum proportion of nitrogen from solid manure to soil | [0-1] |
| tnminmx |  | Maximum proportion of organic nitrogen in mineralization process | [0-1] |
| tnorgmx |  | Maximum proportion of nitrogen from passive to active organic matter | [0-1] |

S11B Table Parameters of nitrogen cycle namespace.

| com | npla | nres | norgl | norg | nmin | tndescd | tndescdl | tnorgmx | tndesch | tnminmx | prtvm | prvolatn | nestie |
| --- | --- | --- | --- | --- | --- | --- | --- | --- | --- | --- | --- | --- | --- |
| Calluna (Calluna vulgaris) | 0.01 | 0.5 | 500 | 1 | 0.5 | 0.001 | 0.007 | 0.000025 | 0.5 | 0.01 | 0.6 | 0.5 | 5 |
| FestucaRubra (Agrostis curtisii) | 0.02 | 0.5 | 500 | 1 | 0.5 | 0.02 | 0.001 | 0.00005 | 0.5 | 0.02 | 0.5 | 0.5 | 5 |
| Helictotrichon (Helictotrichon cantabricum) | 0.01 | 0.5 | 500 | 1 | 0.5 | 0.005 | 0.004 | 0.00005 | 0.5 | 0.01 | 0.5 | 0.5 | 5 |
| Phragmites | 0.01 | 0.5 | 500 | 1 | 0.5 | 0.005 | 0.004 | 0.000025 | 0.5 | 0.01 | 0.5 | 0.5 | 5 |
| Polypodiopsida | 0.01 | 0.5 | 500 | 1 | 0.5 | 0.005 | 0.004 | 0.000025 | 0.5 | 0.01 | 0.6 | 0.5 | 5 |
| Senecio (sp) | 0.02 | 0.5 | 500 | 1 | 0.5 | 0.01 | 0.003 | 0.00005 | 0.5 | 0.01 | 0.5 | 0.5 | 5 |
| UlexGallii | 0.02 | 0.5 | 500 | 1 | 0.35 | 0.005 | 0.004 | 0.00005 | 0.5 | 0.01 | 0.5 | 0.5 | 5 |
| Carex | 0.02 | 0.5 | 500 | 1 | 0.5 | 0.01 | 0.003 | 0.00005 | 0.5 | 0.01 | 0.5 | 0.5 | 5 |
| Gramineae | 0.02 | 0.5 | 500 | 1 | 0.5 | 0.02 | 0.001 | 0.00005 | 0.5 | 0.02 | 0.5 | 0.5 | 5 |
| UlexEuropaeus | 0.02 | 0.5 | 500 | 1 | 0.5 | 0.005 | 0.004 | 0.00005 | 0.5 | 0.01 | 0.5 | 0.5 | 5 |

S11C Table Equations of nitrogen cycle namespace.

| id | time (t=>0) | |
| --- | --- | --- |
|  | **t=0** | **t>0** |
| descd | ft * fh * tndescd * biod | |
| descdl | ft * fh * tndescdl * biod | |
| fn | a =  ftrh*xf*ph1-rar  *fn=*  if (ftrh*ph==0){  0  }else{  crec+crecra | |
| nbiod | biod*npla*prtvm | nbiod + ndesf - ndescd - ndescdl |
| nbiodr | biod * npla * prtvm | nbiodr + ndesfr - ndescdr – ndescdrl |
| nbioh | 0 | nbioh + nhgm2ift - ndesch |
| ndescd | ft * fh * tndescd * nbiod | |
| ndescdl | ft * fh * tndescdl * nbiod | |
| ndescdr | ft * fh * 1.5 * tndescd * nbiodr | |
| ndescdrl | ft * fh * 1.5 * tndescdl * nbiodr | |
| ndesch | ft * fh * tndesch * nbioh | |
| ndesf | *pi =*  if (biov == 0){  0  }else{  ivgm2ift/biov  *dt =*  if pi<pimn {  fmn  } else if pi>pimx {  fmx  } else {  fmn+(((fmx-fmn)/(pimx-pimn))*(pi-pimn)) }  ndesf =dt*biom*npla*prtvm) + ((1-ksv)*svgm*npla) + ((1-ksm)*smgm*npla*prtvm) | |
| ndesfr | dr * biomr * npla * prtvm | |
| nmin | nmin (S11B Table) | *den =*  nres + nreabs + nmin + nminer + (nogm2ift * (1 - prvolatn)) – nperc    *nminc =*  nmin + nminer + nogm2ift * 1 - prvolatn- nperc - nmin + nminer + nogm2ift * 1 - prvolatn- npercden*crec+crecr*npla  *nmin=*  if (nminc < 0){  0  }else{  nminc |
| nminer | ft * fh * tnminmx * norg | |
| norg | norg (S11B Table) | if (nestie is unknown){  norg + ndescd + ndescdr + norgan + ndesch - nminer  }else{  norg + ndescd + ndescdr + norgan + ndesch - nminer + nestie |
| norgan | ft * fh * tnorgmx * norgl | |
| norgl | norgl (S11B Table) | norgl+ ndescdl + ndescdrl – norgan |
| nperc | *perc=*  if (dri < 0){  -dri  }else{  0  *nperc* =  nmin +percperc + adt | |
| nreabs | (senes + senesr) * prtvm * npla | |
| nres | nres (S11B Table) | *den =*  nres + nreabs + nmin + nminer + (nogm2ift * (1 - prvolatn)) – nperc  *nresc=*  nres + nreabs - nres + nreabsden*crec + crecr* npla  *nmin=*  if (nresc < 0){  0  }else{  nresc |
| nvolat | nogm2ift * prvolatn | |

**S2 Appendix**

Comparison between the Puerto results processed by a GIS expert (S12A Fig) with the results that can be obtained by a basic PaL user (S12B Fig). In contrast to PaL, Puerto results:

- are aggregated by plot identifier (S12 Table),
- are unaware of the distribution of vegetation,
- do not distinguish areas without palatable vegetation within each plot and
- are not spatially explicit (in some cases, a GIS expert can link results from Puerto tables to its location by plot identifier).

One thing that is particularly striking is that the low vegetation growth values in the Puerto example (S12A Fig) are an artifact of taking the mean of vegetation growth on a pastoral unit which has significant bare areas or areas with non-palatable vegetation (grey area in S12B Fig).
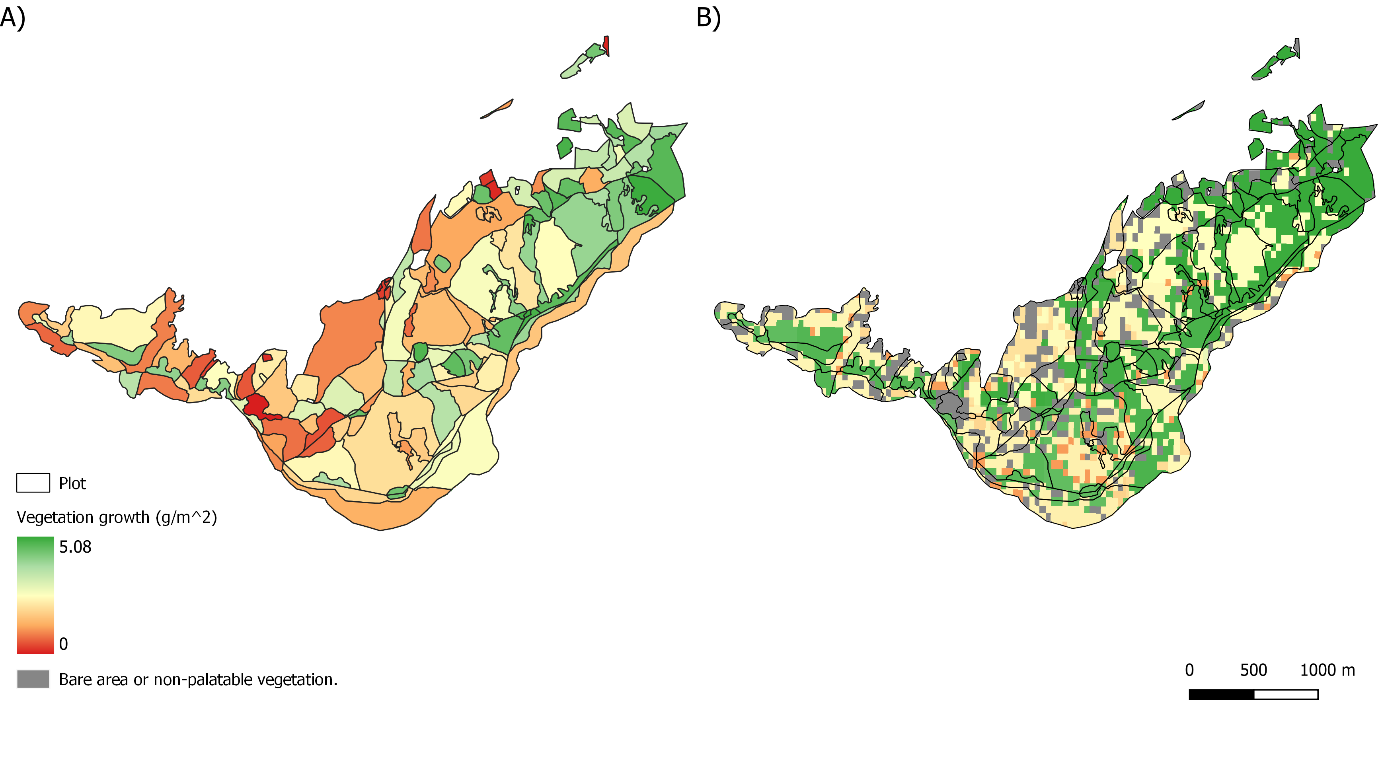


S12 Fig. Results of Vegetation Growth (g/m^2^). A) Puerto and B) PaL results.

Below, we compare the distribution of both PaL and Puerto result for vegetation growth through a box plot (S13 Fig). In order to compare Puerto and PaL results, we calculate the mean vegetation growth by plot in the PaL results. Moreover, S14 Fig tracks vegetation growth results in increasing order over the plots (S12 Table).


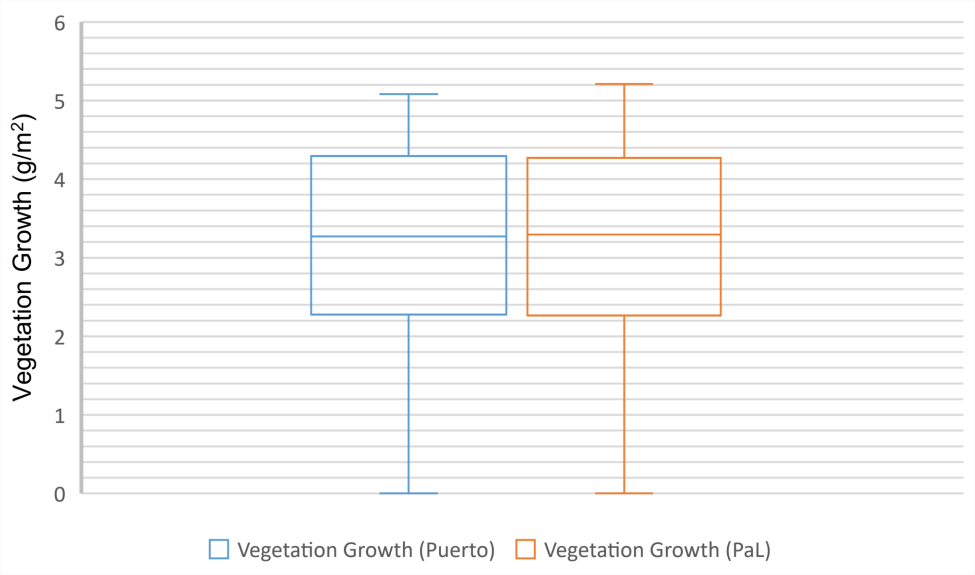


S13 Fig. Box plot comparing distribution of vegetation growth results by plot for Puerto and PaL.


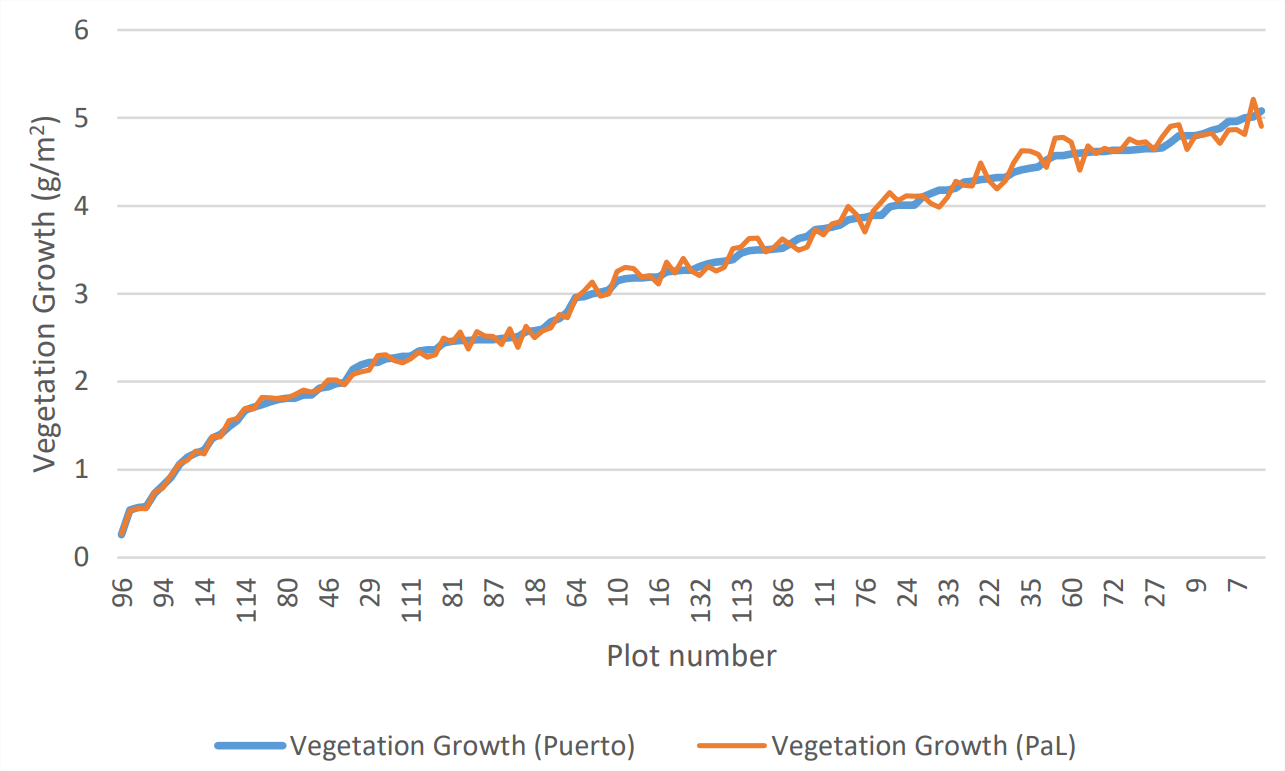


S14 Fig. Line graph wit PaL and Puerto vegetation growth results by plot.

S12 Table Results of vegetation growth (g/m^2^) in Puerto and PaL by each plot in the same context.

| Plot | Vegetation Growth (Puerto) | Vegetation Growth (PaL) |
| --- | --- | --- |
| 1 | 4.64 | 4.71 |
| 2 | 1.94 | 2.02 |
| 3 | 1.74 | 1.82 |
| 4 | 4.57 | 4.77 |
| 5 | 1.99 | 1.97 |
| 6 | 0.54 | 0.53 |
| 7 | 4.96 | 4.86 |
| 8 | 4.8 | 4.92 |
| 9 | 4.8 | 4.64 |
| 10 | 3.04 | 3.00 |
| 11 | 3.73 | 3.73 |
| 12 | 3.89 | 3.94 |
| 13 | 4.61 | 4.68 |
| 14 | 1.19 | 1.21 |
| 15 | 4.66 | 4.78 |
| 16 | 3.19 | 3.20 |
| 17 | 4.63 | 4.62 |
| 18 | 2.57 | 2.63 |
| 19 | 1.68 | 1.70 |
| 20 | 3.17 | 3.30 |
| 21 | 4.8 | 4.79 |
| 22 | 4.3 | 4.49 |
| 23 | 4.1 | 4.12 |
| 24 | 4.01 | 4.06 |
| 25 | 3.78 | 3.82 |
| 26 | 2.22 | 2.13 |
| 27 | 4.65 | 4.73 |
| 28 | 4.82 | 4.81 |
| 29 | 2.19 | 2.11 |
| 30 | 3.84 | 4.00 |
| 31 | 2.27 | 2.24 |
| 32 | 4.27 | 4.24 |
| 33 | 4.18 | 3.98 |
| 34 | 2.49 | 2.42 |
| 35 | 4.41 | 4.63 |
| 36 | 4.18 | 4.10 |
| 37 | 4.32 | 4.19 |
| 38 | 4.59 | 4.72 |
| 39 | 2.51 | 2.39 |
| 40 | 3.25 | 3.36 |
| 41 | 2.6 | 2.58 |
| 42 | 4.01 | 4.12 |
| 43 | 1.85 | 1.90 |
| 44 | 2.36 | 2.28 |
| 45 | 2.48 | 2.57 |
| 46 | 1.93 | 1.92 |
| 47 | 4.63 | 4.63 |
| 48 | 4.6 | 4.41 |
| 49 | 3.36 | 3.26 |
| 50 | 4.63 | 4.76 |
| 51 | 1.49 | 1.56 |
| 52 | 3.15 | 3.25 |
| 53 | 2.36 | 2.31 |
| 54 | 3.65 | 3.53 |
| 55 | 3.46 | 3.53 |
| 56 | 3.57 | 3.56 |
| 57 | 3.63 | 3.50 |
| 58 | 0.82 | 0.79 |
| 59 | 0.57 | 0.56 |
| 60 | 4.57 | 4.78 |
| 61 | 5.08 | 4.90 |
| 62 | 4.32 | 4.28 |
| 63 | 4.88 | 4.71 |
| 64 | 2.79 | 2.73 |
| 65 | 2.97 | 3.03 |
| 66 | 2.35 | 2.34 |
| 67 | 3.34 | 3.31 |
| 68 | 3.87 | 3.70 |
| 69 | 3.5 | 3.63 |
| 70 | 3.18 | 3.29 |
| 71 | 4.62 | 4.60 |
| 72 | 4.62 | 4.65 |
| 73 | 2.72 | 2.76 |
| 74 | 1.98 | 2.02 |
| 75 | 4.14 | 4.03 |
| 76 | 3.86 | 3.90 |
| 77 | 3.27 | 3.40 |
| 78 | 3.49 | 3.63 |
| 79 | 2.47 | 2.56 |
| 80 | 1.8 | 1.81 |
| 81 | 2.44 | 2.50 |
| 82 | 4.28 | 4.23 |
| 83 | 4.52 | 4.44 |
| 84 | 1.71 | 1.69 |
| 85 | 1.22 | 1.18 |
| 86 | 3.51 | 3.53 |
| 87 | 2.48 | 2.52 |
| 88 | 3.19 | 3.11 |
| 89 | 0.58 | 0.56 |
| 90 | 3.89 | 4.04 |
| 91 | 3.52 | 3.62 |
| 92 | 2.47 | 2.37 |
| 93 | 0.91 | 0.93 |
| 94 | 0.73 | 0.74 |
| 95 | 1.14 | 1.11 |
| 96 | 0 | 0.00 |
| 97 | 1.81 | 1.81 |
| 98 | 3.76 | 3.80 |
| 99 | 0.26 | 0.27 |
| 100 | 3 | 3.13 |
| 101 | 2.22 | 2.30 |
| 102 | 1.06 | 1.07 |
| 103 | 4.44 | 4.58 |
| 104 | 3.26 | 3.23 |
| 105 | 3.02 | 2.97 |
| 106 | 1.4 | 1.37 |
| 107 | 2.68 | 2.61 |
| 108 | 1.77 | 1.82 |
| 109 | 3.99 | 4.15 |
| 110 | 4.38 | 4.49 |
| 111 | 2.29 | 2.22 |
| 112 | 1.81 | 1.85 |
| 113 | 3.39 | 3.51 |
| 114 | 1.56 | 1.58 |
| 115 | 2.5 | 2.60 |
| 116 | 1.85 | 1.88 |
| 117 | 2.14 | 2.08 |
| 118 | 3.18 | 3.19 |
| 119 | 1.36 | 1.38 |
| 120 | 3.5 | 3.47 |
| 121 | 4.31 | 4.29 |
| 122 | 4.01 | 4.11 |
| 123 | 4.65 | 4.64 |
| 124 | 4.2 | 4.28 |
| 125 | 5 | 4.81 |
| 126 | 4.96 | 4.87 |
| 127 | 5.01 | 5.21 |
| 128 | 2.29 | 2.26 |
| 129 | 4.43 | 4.62 |
| 130 | 2.96 | 2.95 |
| 131 | 4.72 | 4.91 |
| 132 | 3.27 | 3.26 |
| 133 | 3.37 | 3.30 |
| 134 | 2.58 | 2.50 |
| 135 | 2.26 | 2.31 |
| 136 | 3.31 | 3.21 |
| 137 | 3.74 | 3.67 |
| 138 | 2.46 | 2.45 |
| 139 | 2.48 | 2.52 |
| 140 | 4.86 | 4.83 |
