# Supplementary material for: Scientific modelling can be accessible, interoperable and user friendly: A case study for pasture and livestock modelling in Spain
Source: PLoS One. 2023 Feb 24;18(2):e0281348. doi: 10.1371/journal.pone.0281348 (PMC9957615; doi:10.1371/journal.pone.0281348)
Supplement: Attachment — Submitted filename: Response to Reviewers.docx [file pone.0281348.s016.docx]

Additional Editor Comments:

1. Please amend either the abstract on the online submission form (via Edit Submission) or the abstract in the manuscript so that they are identical.

We thank the Editor for their time and suggestion. We uploaded the suggested changes in the online submission.

2. We note that Figures 1, 2, 6, 7, 9, 10 and 11 in your submission contain map images which may be copyrighted. All PLOS content is published under the Creative Commons Attribution License (CC BY 4.0), which means that the manuscript, images, and Supporting Information files will be freely available online, and any third party is permitted to access, download, copy, distribute, and use these materials in any way, even commercially, with proper attribution. For these reasons, we cannot publish previously copyrighted maps or satellite images created using proprietary data, such as Google software (Google Maps, Street View, and Earth). For more information, see our copyright guidelines: <http://journals.plos.org/plosone/s/licenses-and-copyright>.

We require you to either (1) present written permission from the copyright holder to publish these figures specifically under the CC BY 4.0 license, or (2) remove the figures from your submission:

 a. You may seek permission from the original copyright holder of Figures 1, 2, 6, 7, 9, 10 and 11 to publish the content specifically under the CC BY 4.0 license. 

We recommend that you contact the original copyright holder with the Content Permission Form (<http://journals.plos.org/plosone/s/file?id=7c09/content-permission-form.pdf>) and the following text:

“I request permission for the open-access journal PLOS ONE to publish XXX under the Creative Commons Attribution License (CCAL) CC BY 4.0 (<http://creativecommons.org/licenses/by/4.0/>). Please be aware that this license allows unrestricted use and distribution, even commercially, by third parties. Please reply and provide explicit written permission to publish XXX under a CC BY license and complete the attached form.”

Please upload the completed Content Permission Form or other proof of granted permissions as an "Other" file with your submission.

In the figure caption of the copyrighted figure, please include the following text: “Reprinted from [ref] under a CC BY license, with permission from [name of publisher], original copyright [original copyright year].”

 b. If you are unable to obtain permission from the original copyright holder to publish these figures under the CC BY 4.0 license or if the copyright holder’s requirements are incompatible with the CC BY 4.0 license, please either i) remove the figure or ii) supply a replacement figure that complies with the CC BY 4.0 license. Please check copyright information on all replacement figures and update the figure caption with source information. If applicable, please specify in the figure caption text when a figure is similar but not identical to the original image and is therefore for illustrative purposes only.

The following resources for replacing copyrighted map figures may be helpful:

 USGS National Map Viewer (public domain): <http://viewer.nationalmap.gov/viewer/>

The Gateway to Astronaut Photography of Earth (public domain): <http://eol.jsc.nasa.gov/sseop/clickmap/>

Maps at the CIA (public domain): <https://www.cia.gov/library/publications/the-world-factbook/index.html> and <https://www.cia.gov/library/publications/cia-maps-publications/index.html>

NASA Earth Observatory (public domain): <http://earthobservatory.nasa.gov/>

Landsat: <http://landsat.visibleearth.nasa.gov/>

USGS EROS (Earth Resources Observatory and Science (EROS) Center) (public domain): [http://eros.usgs.gov/#](http://eros.usgs.gov/)

Natural Earth (public domain): <http://www.naturalearthdata.com/>


Please provide your response in the "Author Comments" box section.

We made the suggested changes in the manuscript. The basemap used in the Figures has different copyright, we add the text required in the figure caption and references part. We describe more detailed information below:

- **Figures 1, 6, 7, 9 and 10 (changed to Fig. 11)**
  - **Data source**: [*https://www.earthenv.org/DEM.html*](https://www.earthenv.org/DEM.html)
  - **Licence**: “EarthEnv Digital Elevation Model Version 1 by [Natalie Robinson and the NCEAS Environment and Organisms (ENO) Working Group](https://www.earthenv.org/DEM.html) is licensed under a [Creative Commons Attribution 4.0 International License](https://creativecommons.org/licenses/by/4.0/).
    Permissions beyond the scope of this license may be available at [http://www.earthenv.org/DEM.html](https://www.earthenv.org/DEM.html). Users should cite [Robinson et al. (2014)](https://www.sciencedirect.com/science/article/pii/S0924271613002360) in the creation of any reports, publications, new data sets, derived products, or services resulting from the use of this data set, and if possible link web pages to the EarthEnv-DEM90 website ([*http://www.earthenv.org/DEM.html*](https://www.earthenv.org/DEM.html)). Please refer to acknowledgements in [Robinson et al. (2014)](https://www.sciencedirect.com/science/article/pii/S0924271613002360) for funding and logistic support for EarthEnv-DEM90”.
- **Figure 2:**
  - **Data source**: *https://www.gadm.org/license.html*
  - **Licence**: “The data are freely available for academic use and other non-commercial use. Redistribution or commercial use is not allowed without prior permission.
    Using the data to create maps for publishing of academic research articles is allowed. Thus you can use the maps you made with GADM data for figures in articles published by PLoS, Springer Nature, Elsevier, MDPI, etc. You are allowed (but not required) to publish these articles (and the maps they contain) under an open license such as CC-BY as is the case with PLoS journals and may be the case with other open access articles. Data for the following countries is covered by a a different license Austria: Creative Commons Attribution-ShareAlike 2.0 (source: Government of Ausria).”
- **Figure 11 (changed to Fig.12)**
  - **Data source:** [*https://openlayers.org/*](https://openlayers.org/)
  - **Licence: “**OpenLayers has been developed to further the use of geographic information of all kinds. It is completely free, Open Source JavaScript, released under the 2-clause BSD License (also known as the FreeBSD).”

3. We note that your Data Availability statement states the following: "All relevant data are within the manuscript and its Supporting Information files."

We also note that you have included two "Data Review URL" external links in your submission:

- <https://zenodo.org/record/6786430#.Ys8MoHbMI2w>
- <https://zenodo.org/record/6786419#.Ys8Mp3bMI2w>

Before we can proceed, please clarify if any of your minimal dataset is located at the provided links.

We will update your Data Availability Statement based on the information you provide.

The dataset used in PaL model is in the code, inside the resources folder. All the data has been generated by us. (<https://zenodo.org/record/6786430#.Ys8MoHbMI2w>).

# <https://zenodo.org/record/6786430#.Ys8MoHbMI2w> link (PaL models). Set of the models working in k.LAB as a result of the scientific research developed in the manuscript.

- <https://zenodo.org/record/6786419#.Ys8Mp3bMI2w> link (Puerto model). Base model on which we have based to adapt it to the PaL semantic model in k.LAB. Model developed by PhD. Juan Busqué with open license.

According to the review process and results, my current attitude is that the manuscript is on the rim of rejection due to three major criticisms. 1. The manuscript is more like a technical report than an academic paper. Actually academic paper should have theoretical highlights to enlighten further advancements. 2. There are faults in presentation. 3. What is the target reader community?

We thank the editor and the reviewers for their time and suggestions. We address the 3 points in our responses to reviewers’ comments and below.

1. With this article we have strived not to simply report on a specific agricultural model, but to highlight more general and more scientifically relevant technical problems related to **reusability** and **FAIRness** in models. This a key issue especially in this period of history, in which we are living a **crisis of scientific reproducibility**, recognized in many publications, including some in PLoS.

- Sandve, G. K., Nekrutenko, A., Taylor, J. & Hovig, E. Ten Simple Rules for Reproducible Computational Research. PLoS Comput. Biol. 9, e1003285 (2013).
- González-Beltrán, A. et al. From Peer-Reviewed to Peer-Reproduced in Scholarly Publishing: The Complementary Roles of Data Models and Workflows in Bioinformatics. PLoS ONE 10, e0127612 (2015).

2. For this reason, our paper spends quite a bit of effort illustrating the semantic integration technique used to transform a monolithic and hard-to-use model into a modular, readable and easy to validate formalism that has the potential of changing agricultural and socio-ecological modelling much beyond the specific model discussed. Getting the overall aim of our work through to specialist reviewers has been difficult, no doubt because of our own shortcoming in making it as clear as possible. With this revision, we trust we have corrected those faults. At the same time, we have made all possible efforts to make the original Puerto model more accessible and documented, as per comments of one reviewer. We also hope that the English-language description of Puerto improves transparency, not without noting a subtle, but clearly visible, bias against science published in non-English literature.

3. Specifically, our effort to clarify the target community and overall scope of the work didn’t come out straightforwardly for one reviewer. Originally, the target community was implied in the last lines of the original abstract:

“We argue that this work demonstrates key steps needed to create more Findable, Accessible, Interoperable and Reusable (FAIR) models. This is particularly essential in environments as complex as agricultural systems, where multidisciplinary knowledge needs to be integrated across diverse spatial and temporal scales in order to understand complex and changing problems.”

In the revision, the introduction provides added clarity and purpose: “The aim is to showcase to the “social-ecological” modelling community the delivery of an existing, monolithic model, into a more modular, transparent and accessible approach to potential end users, regional managers, farmers and other stakeholders.”

**Comments to the Author**

**Reviewer #1:** This paper focuses on accessible, interoperable, and user-friendly scientific modeling. Its work is very valuable. However, some comments and suggestions that I hope would help perfecting the manuscript. My comments and suggestions are as follows.

1. Abstract, “The original model, named Puerto, is developed in the R language and includes 1,491 lines of code divided into 13 script files and linked to 19 input tables.” the sentence does not reflect the existing problem that arise, the significance of the study should be highlighted.

We thank the Reviewer for their time and suggestion. We made the suggested changes in the manuscript (lines: 22-24 in the manuscript and 25-29 in the revised manuscript with trach changes).

2. This study mainly shows the advantages of spatial visualization of the PaL model output. Is the reliability of the output results verified? for example, compare the reliability of PaL versus Puerto output. After all, the accuracy of the model is the primary purpose of the analysis.

Indeed, spatialization is a significant advantage of the approach described in the article, which also makes the results not directly comparable for accuracy. However, to respond to your very good point, we have run a comparison exercise in Supplementary Information Appendix 2. For this we focus on the PaL vegetation growth model in the area that Puerto uses as teste case and that is provided in an online repository with the code (<https://doi.org/10.5281/zenodo.6786419>). Fig 1 shows the Puerto example location compared to our case study.

Fig 1. Location of PaL case study and the area that Puerto has as example in their code.

3. Why was May chosen for modelling?

We decided to initialize the model in May 2018 because it was the starting date of the vegetation distribution map, according to our data availability. Other resources that we used in PaL models are related to weather, topography or livestock distribution and did not have the same limitations (lines: 297-302 in the manuscript and 320-325 in the revised manuscript with trach changes).

4. Fig. 9, I tried but really cannot understand why livestock mass variation of is negative?

At the date that the model was generated, the estimation of pasture availability (Supporting Information SA1, S5) was lower than the estimation for the energy needs of the livestock present in the plot (Supporting Information SA1, S10), that is why they have a decrease in weight. Energy needs are related to metabolism, the type of terrain they are on and the animal condition, among other things. In addition, it must be taken into account that there are cows and mares in the same field, increasing the competition for food. It is a useful tool that can be used by the farmer to know if the number of livestock and the forage production (carrying capacity) are balanced. Lines: 393-400 in the manuscript and 428-435 in the revised manuscript with trach changes.

For example, the following graph shows the variation of weight in different cattle livestock groups during one year. We also decided to add this figure to the article as it may help to better understand this point.

5. The Pal model takes into account the vegetation growth cycle, changes in livestock quality, nitrogen cycle, etc. However, I don't know how to achieve the best economic optimal benefits in the output results, which may be more desired by users, and more meaningful.

We haven’t included yet an optimization algorithm to respond to your questions and we regard this work as an important future development. The modular semantic environment indeed provides a way to include this module without any modification to the overall model. Nevertheless, an end user can decide how to manage their pastures depending on the results of the model. For example, the land manager can explore if the livestock is losing weight, if the vegetation is growing or if he needs to add more fertilizer or cut the grass, among others.

In the future, we expect to add a model that calculates whether the pasture is sustainable or not, based on the carrying capacity, fertilizer or type of vegetation. This model would be based on scientific studies:

- Holechek J, Pieper RD, Herbel CH. Range Management: Principles and Practices. 6th ed. Pearson Education; 2011. 456 p.
- Elgersma A, Struik PC, Maesen LJG van der. Grassland science in perspective. [Internet]. Wageningen Agricultural University; 1996. Available from: https://research.wur.nl/en/publications/grassland-science-in-perspective

We have included this in the article as future developments in lines: 572-574 in the manuscript and 614-616 in the revised manuscript with trach changes.

6. The reference format is very bad, please check each reference carefully.

We made the suggested changes in the manuscript (lines: 589-836 in the manuscript and 637-883 in the revised manuscript with trach changes).

**Reviewer #2**: This is a work that purports to link pasture and livestock modeling with GIS in a manner that would be both user-friendly and suitable for land policy making. The authors are advised to consider the following:

a) The whole work is more likely to be useful as a technical report than a scientific paper. Although it has undeniably been written in a scientific manner, its scope of readership is restricted to scientific officers of the geographical area it refers to or to people involved in making livestock modeling software more user-friendly. I am not sure it is possible to make more appealing to a wider readership, but it certainly has to.

We thank the reviewer for their time and suggestions, which we believe have improved the quality and relevance of the manuscript for a wider audience. We address the broader issue of the study’s scope and wider relevance in our responses to comments B, C, D, and below.

As explained above, we wrote this article to illustrate a more efficient way to write, deliver and share models, not merely to discuss a rewrite of an existing model. After clarifying this further in our revision, we regard PLoS ONE as an appropriate outlet due to its generalist readership, and its ability to publish even technical pieces. We appreciate that the reviewer’s comments made us clarify our target audience which perhaps was implicit in the abstract: now we added “The aim is to showcase to the “social-ecological” modelling community the delivery of an existing, monolithic model, into a more modular, transparent and accessible approach to potential end users, regional managers, farmers and other stakeholders.”

Please see our responses below, also covering the issue of geographical scope.

b) This is largely due to the fact that it shows how render a cumbersome existing software named “Puerto" into a more friendly and effective one. One problem is that “Puerto” is by far and largely unknown to experts. And the only documentation offered here about it here are four references: two papers by Busque, one by Bedia et al and one by Marcos et al. Of these papers, three are in Spanish and one in English, but only as a conference paper. In short, the reader can not access this system, and if one does not speak Spanish, is unable not gain adequate knowledge about it.

We agree that this specific example is a locally developed and used model, with most of its documentation available in non-English languages.

Even though it has been used in real life by practitioners in Cantabria, references 38 and 47-49), Puerto’s (and other models’) accessibility is one dimension of the problems this paper addresses. We have also clarified how Puerto is not inventing anything, but using equations which are well established in the scientific literature (references 39-46), which we now provide as supplementary information (Supporting Information, S1 Appendix).

Additionally, regarding the geographical scope, it must be noted that the work described in this article has made it possible to apply the implemented model in the four case studies of the ALICE project, which covers the Atlantic space much beyond northern Spain (Portugal, France and Northern Ireland).

At the same time Puerto is just an example that resonate with many scientific models written for research purposes. Most of them end up published in an article and then forgotten. Some are used with “local significance”. Very few become internationally recognized. This article is about making all scientific knowledge more usable.

c) This is also due to the rather weak presentation of this system (“Puerto") in the paper, so the reader can not appreciate (at least to the extent that the authors would like) the significance of their effort to improve it by creating PaL etc. And, in any case, the paper gives the impression that the conversion of “Puerto" to "PaL" and its equivalents is of local significance only (for North-Western Spain).

We believe we have improved the presentation of Puerto and elaborated further on the issue of “non-local significance”. As described in the response to comment B above, we have more comprehensively described Puerto, its uses, and its relevance for modelling both within and beyond Spain.

d) Consequently, converting a local system to a new one so as to become more useful or user-friendly is of very limited interest to the journal’s readership.

By revising the paper as described above to clarify that its novel contribution is the conversion of Puerto to PaL within the globally used ARIES system, we highlight the general path toward making models interoperable and the value of that for scientific users, beyond the application of a particular model.

e) Figure 15 displays the funding source (“Alice") only and does not elucidate the reader in what has been done.

We made the suggested changes in the manuscript (lines: 111-115 in the manuscript and 119-123 in the revised manuscript with trach changes).

f) Figure 8: There should be a unified legend explaining what is displayed by the figure. The legend is split in two: one in English and one in Spanish above the figure.

We made the suggested changes in the manuscript (lines: 370-380 in the manuscript and 403-413 in the revised manuscript with trach changes).

g) Table 3: It is unclear what is t=1 all throughout the table (what does t stand for?) and if it takes the same value everywhere then why bother about it?

Table 3 is the first five lines of “Vegetation Limiting Factor” internal table of Puerto model in R. “t” is counting the daily time-step in the timeline meaning “t=1” the first day in the dynamic model. We made the changes in the manuscript (lines: 327-342 in the manuscript and 350-365 in the revised manuscript with trach changes).

h) Figures 13 and 14 are not explained adequately in the legend, particularly the window c at the down right part of the figure.

Figures 13 and 14 have changed to 14 and 15 respectively. We made the suggested changes into the manuscript (lines: 439-448 in the manuscript and 479-489 in the revised manuscript with trach changes).

i) The title of the paper needs to change so as to correspond to the content of the paper: this is a local study aiming at improving a local software system so as to become more efficient and user-friendly. This does not justify the use of a general title like the one that has been used. And, certainly, since this is a local study, it should be indicated in the title also: the word “Spain” should appear somewhere (or any other indicating the geographical location that the study concerns).

We already responded on the non-local significance of the case study. The article is about making scientific models (often developed on case by case demand, often from scratch), more available as modular components, to be integrated in other computational workflows and help build up collective knowledge about human-natural integrated systems.

While Puerto can be defined as a “local” software, K.LAB itself is definitely not a local software. ARIES (powered by k.LAB) is endorsed by the UN DESA for natural capital accounting, mostly for its approach to interoperability. More information available at <https://seea.un.org/content/aries-for-seea> or in this recent publication:

- Balbi, S., Bagstad, K.J., Magrach, A. et al. The global environmental agenda urgently needs a semantic web of knowledge. Environ Evid 11, 5 (2022). <https://doi.org/10.1186/s13750-022-00258-y>

We made sure to clarify the scope of this technology. Nevertheless we agree with you that the title is too generic, thus we proposed a new one: *“*Scientific modelling can be accessible, interoperable and user friendly: A case study for pasture and livestock modelling in Spain”.

j) The references might need some updating, as the most recent one is from 2020, and we are already running the year 2022.

We made the suggested changes into the manuscript (references: 57-60, 66).

**Reviewer #3:** The article presents an adaptation of the existing model Puerto to an improved version that is more accessible, interoperable and user friendly, and could thus be of greater use for agriculture stakeholders in the face of future challenges for the sector. The article is appropriate for publication in Plos One, however I do have a list of minor comments and suggestions on the main manuscript and the supporting information mostly aiming to improve clarity.
There was no page or line numbering on the manuscript which makes it difficult to comment.

We thank the Reviewer for his/her time and suggestion. We added line numbering in the manuscript.

Below I’m using the page number of the submitted document and the related paragraph.

- P9, par1: ‘one main issues’-> of the

We made the suggested changes in the manuscript (lines: 71 in the manuscript and 78 in the revised manuscript with trach changes).

- P9, par2: ‘growth, senescence death and litterfall’-> death?

We made the changes in the manuscript (lines: 84 in the manuscript and 91 in the revised manuscript with trach changes).

- P10, par1: you are mentioning novel approaches, yet the most recent citation is almost a decade old. Try to incorporate recent advances in the field on this and/or related domains. This is also something that is missing from the discussion.

We made the suggested changes in the manuscript (references: 56-60).

- P13, par2: remove ‘with’

We made the suggested changes in the manuscript (lines: 169 in the manuscript and 179 in the revised manuscript with trach changes).

- P13, par2: Please check syntax of the last sentence.

We made the suggested changes in the manuscript (lines: 173-175 in the manuscript and 184-187 in the revised manuscript with trach changes).

- P18, par4: three main models are stated but four are described (moisture, radiation, temperature, nitrogen). Also, how are extreme events defined and quantified?

We made the suggested changes in the manuscript (lines: 304 in the manuscript and 328 in the revised manuscript with trach changes). Extreme events are not quantified itself, but are implicit in some of the models. As weather data is an input of some models related to vegetation and, each type of vegetation has limiting vital parameters (minimum and maximum temperature, radiation and humidity to live), any event outside these vital parameters will influence the outcome of several models, as the vegetation will not be able to grow or will die. For example, some models that are influenced directly or indirectly are the vegetation growth model, vegetation death or livestock ingestion, among others.

- Figure 6. It’s not clear how these values are computed. Are the default values maintained and in each graph a different limitation is applied? At what values of the limiting factor and what are the units?

The model values that limit the growth of vegetation are between 0 and 1. These values have no units as it is an index that is created after several calculations (Supporting Information S1 Appendix: S2-4 Fig and S2-4 A-C Table). It is calculated taking into account whether the environmental conditions affecting the vegetation (temperature, radiation, humidity and nitrogen) are sufficient for it to grow. If the vegetation is able to reach its growth needs through the inputs from the received environmental conditions, it will grow the maximum possible [Ratio of limiting factor for vegetation growth = 1], equalling the actual growth to the potential growth. If, on the other hand, environmental conditions are adverse, the "Ratio of limiting factor for vegetation growth" will be smaller than 1, reaching 0 when there is no growth. Lines: 315-318 in the manuscript and 338-341 in the revised manuscript with trach changes.

- Figure 7 You point out differences between maximum potential and maximum actual growth. This could be better visualized if the same color range was applied on both graphs. If the range was 0-5.29g on both graphs then we’d see a shift of B graph towards yellow/red.

We made the suggested changes in the manuscript (lines: 359-362 in the manuscript and 385-388 in the revised manuscript with trach changes).

- Figure 8: Caption needs to be more informative and/or translate the Spanish title (and axes). Lines are ingestion and bars vegetation? What do the error bars stand for?

We made the suggested changes in the manuscript (lines: 370-380 in the manuscript and 403-413 in the revised manuscript with trach changes).

- Figure 9: Consider the same color range suggestion as figure 7. Also, why is the livestock mass decreasing? Maybe that’s worth commenting on.

We change the color in the manuscript (lines: 388 in the manuscript and 423 in the revised manuscript with trach changes). The model has been generated for time 1, the livestock have not yet been able to eat enough to compensate for the energy they need, so the weight is negative. They lose more weight in areas with steeper slopes, where less vegetation grows and the effort to move is greater. Moreover, for this case study, in the majority of plots, cows and mares coexist, creating a greater competition between them.

- Table 3: please inset units for the parameters.

The parameters in the Table 3 have no units. “IDMancha”, “com”, “com2” and “t” are qualitative (the numbers are codes related to the variables). The parameters “FT”, “FR”, “FH” and “FN” are rankings valued 0 to 1. We clarified this in the manuscript (lines: 327-342 in the manuscript and 350-365 in the revised manuscript with trach changes).

- Figure 13: A description of what A and B is should be provided in the caption

Figure 13 have changed to Figure 14. We made the suggested changes in the manuscript (lines: 439-441 in the manuscript and 479-482 in the revised manuscript with trach changes).

- Supporting information
  - I’ve found the units for many of the described variables either inconsistent, missing or confusing. Please carefully go through the whole list and check for inconsistencies and complete missing information. if the same type of variables have indeed different units please clarify why. Some examples:
  - Proportions: in same cases the range ([0 1]) is given (eg adic, st), some are unitless (eg fhs, cirhift02) and some have units day (eg pmin, pimx).

We made the changes in the Supporting Information S1 Appendix file.

- Mass: shouldn’t all variables related to contents, biomass, mass be in kg (eg ihcift)? Similar for concentrations (eg nogm2ift)?

Puerto has different units for mass, volume or area depending on the variable and transform the units in the code accordingly. For example, in the line of R code from the "Port" model:

, where “VarPesoInd” (livestock weight change) is in grams and PVr (livestock weight) in in kg. In the case of PaL, this calculation is not necessary because k.LAB does the transformation automatically. Therefore, the script in k.IM language is:

- Temperature: some missing (eg st1)

We made the suggested changes in the Supporting Information S1 Appendix file.

- Shouldn’t digestibility be in % (eg digm, digv)?

The values are in fact proportions, that is why we have put [0-1] in units instead of the symbol %.

- Table S9. Almost all units are missing.

We made the suggested changes in the Supporting Information S1 Appendix file.

- For pv weight is given in kg but right below for pvr it has no units.

We made the suggested changes in the Supporting Information S1 Appendix file.

- Table 10. What is MJ/t (eg emi), is it different to MJ/d (eg nmd2)?
- The Puerto manual uses the symbol "t" in order to identify each time step in the model, which in this case it refers to day (in Puerto as "d"). At the time of rewriting and adapting part of the Port manual, we have made a typo error as those symbology is confusing and is not using SI conventions.Energy in some cases it is given, in some omitted (eg enigan, enimd) and in same it is confusing (eg in egkg, if it’s the energy needed to increase 1kg of body weight then the unit should be simply MJ).

We made the suggested changes in the Supporting Information S1 Appendix file. “egkg” means energy needed for increase one kilogram, for this reason the units are MJ/kg.

- Since you are using m^2, and m^3, then volume variables (eg dri, et0) should also be mm^3, not mm.

Milimeters (mm) are describing a density of a volume over area (mm^3^/mm^2^). It is used in incident volumes representing water content on an area as, for example, precipitation over an area; but it cannot represent water content in the soil. In the case of water content in the soil, the unit m^3^/m^3^ is refers to a given volume of water contained in given volume of substrate, as the water content at field capacity or the water content at wilting point^1^. However, k.LAB can corrects and rescales units based on spatial and temporal context in case that there is no consistency between models. (lines: 521-523 in the manuscript and 562-564 in the revised manuscript with trach changes).

^1^FAO: Allen RG, Pereira LS, Raes D, Smith M. Crop evapotranspiration - Guidelines for computing crop water requirements - FAO Irrigation and drainage paper 56. FAO (Food and Agriculture Organization of the United Nation); 1998. Available from: <http://www.fao.org/3/x0490e/x0490e00.htm#Contents>; page 162, equation 82 and 83, where ADT is as TAW and AFA is as RAW.

- Also please check numbering on figure S10

We made the suggested changes in the Supporting Information S1 Appendix file.
