# Supplementary material for: Scientific modelling can be accessible, interoperable and user friendly: A case study for pasture and livestock modelling in Spain
Source: PLoS One. 2023 Feb 24;18(2):e0281348. doi: 10.1371/journal.pone.0281348 (PMC9957615; doi:10.1371/journal.pone.0281348)
Supplement: Attachment — Submitted filename: Response to Reviewers.docx [file pone.0281348.s017.docx]

Review Comments to the Author

Reviewer #4: Torres et al. improve the Puerto model by k.IM language and provide a case study on pasture and livestock modelling in Spain. This work is timely and useful for model users. Because of complexity in model framework and practical application, it would be helpful if the authors can further provide detailed tutorial for end-users, such as user guide or video tutorial. I hope that more modelers will contribute to this model framework based on your nice open-source work. Some specific comments may help improvement of this manuscript.

We thank the reviewer for this suggestion. We added a readme file with the instructions to run the PaL models in k.LAB in the online repository and explained it in the manuscript. (Revised Manuscript with Track Changes: line 183, Manuscript: line 183)

L264-268, please clarify more information on data sources of PaL models, including local measurements and global data. Is raster as the raw measurements or produced by measurement data by scaling up? And, which global databases did you use?

We thank the reviewer for this suggestion. Most of the input data were created within the ALICE project partners (lines 111-117) and it is described in article (1), for instance, the data related to climate and topography. To create the vegetation cover map by species, we validated the biodiversity data and habitat mapping from (1) with data collected by Dr. Juan Busqué from previous projects (2–4). We also used the global soil texture map from the ISRIC (World Soild Information) foundation because we could not develop a local soil map (5). Most of the raster maps were produced through downscaling techniques to improve the spatial resolution. In addition, k.Explorer shows all the data sources used for each model (lines 435-441 and 448-454). We made the suggested changes in the manuscript (Revised Manuscript with Track Changes': lines 266-268, Manuscript: lines 266-268).

1.	Fonseca A, Santos JA, Mariza S, Santos M, Martinho J, Aranha J, et al. Tackling climate change impacts on biodiversity towards integrative conservation in Atlantic landscapes. Global Ecology and Conservation. 2022 Oct 1;38:e02216.

2.	Bedia J, Cabañas S, Busqué J. Productivity and plant diversity are related to a community functional signature in mountain grasslands. In 2009. p. 79–82.

3.	Busqué J. De la investigación a la práctica: herramientas para gestionar la ganadería de montaña y los pastos comunales de Cantabria dentro de la política agraria común. Revista Pastos. 2014 Jun;6(1):6–42.

4.	Busqué J, Fernández N, Fernández B. A decision support tool to design rangeland sustainable grazing systems. Sustainable grassland productivity: Proceedings of the 21st General Meeting of the European Grassland Federation, Badajoz, Spain, 3-6 April, 2006. 2006;682–4.

5.	Batjes NH, Ribeiro E, van Oostrum A. Standardised soil profile data to support global mapping and modelling (WoSIS snapshot 2019). Earth System Science Data. 2020 Feb 10;12(1):299–320.

L280, it is better to clarify the algorithms PaL called. If not, the core of PaL models is unclear and a “black box”.

We made the suggested changes in the S1 Appendix, specifically in the tables named as S”*n”*C Table Equations of “*x”*. We add all references and the page number of each below:

- S2C Table Equations of moisture namespace. Page: 5
- S3C Table Equations of radiation namespace. Page: 8
- S4C Table Equations of temperature namespace. Page: 10
- S5C Table Equations of vegetation growth namespace. Page: 13
- S6C Table Equations of vegetation growth namespace. Page: 15
- S7C Table Equations of litterfall namespace. Page: 17
- S8C Table Equations of ingestion namespace. Page: 23
- S9C Table Equations of excretion namespace. Page: 26
- S10C Table Equations of livestock mass namespace. Page: 30
- S11C Table Equations of nitrogen cycle namespace. Page: 34

L290, to what extent the PaL results can directly match the final Puerto outputs? How can we evaluate it? Did you have direct comparison?

We tackled this in the S2 Appendix (Supporting Information, Pages 36-41). We compared the results of Puerto and PaL for the same area, the methodology and the respective limitations.

L315, how did you calculate the ratio of limiting factors for vegetation growth?

We described the calculations in Appendix S1 (Page 8), in the table *S3C Table Equations of radiation namespace*, in the row with the identifier (*id*) named *ftrh*. We clarified in the manuscript (Revised Manuscript with Track Changes: lines 306-309, Manuscript: lines 306-309).

L326 and L407, I cannot find Table 3.

Table 2 was wrongly numbered, thanks for pointing this out (Revised Manuscript with Track Changes: line 330, Manuscript: line 330).

L370, it seems that the legend on the top is not showed in English.

We thank the Reviewer for their time and suggestion. Puerto model displays results as images with legend in Spanish by default. We showed the original Puerto results. We clarified and added the translation in the Figure description (Revised Manuscript with Track Changes: lines 376-382, Manuscript: lines 375-381) as well as in the text (Revised Manuscript with Track Changes: lines 372-373, Manuscript: lines 372-373).
